# Cytotoxicity, anti-angiogenic, apoptotic effects and transcript profiling of a naturally occurring naphthyl butenone, guieranone A

**DOI:** 10.1186/1747-1028-7-16

**Published:** 2012-06-20

**Authors:** Victor Kuete, Tolga Eichhorn, Benjamin Wiench, Benjamin Krusche, Thomas Efferth

**Affiliations:** 1 Department of Biochemistry, Faculty of science, University of Dschang, Dschang, Cameroon; 2 Department of Pharmaceutical Biology, Institute of Pharmacy and Biochemistry, University of Mainz, Staudinger Weg 5, 55128, Mainz, Germany

**Keywords:** Angiogensis, Apoptosis, Cytotoxicity, Guieranone A, Microarray, Pharmacogenomics

## Abstract

**Background:**

Malignant diseases are responsible of approximately 13% of all deaths each year in the world. Natural products represent a valuable source for the development of novel anticancer drugs. The present study was aimed at evaluating the cytotoxicity of a naphtyl butanone isolated from the leaves of *Guiera senegalensis*, guieranone A (GA).

**Results:**

The results indicated that GA was active on 91.67% of the 12 tested cancer cell lines, the IC_50_ values below 4 μg/ml being recorded on 83.33% of them. In addition, the IC_50_ values obtained on human lymphoblastic leukemia CCRF-CEM (0.73 μg/ml) and its resistant subline CEM/ADR5000 (1.01 μg/ml) and on lung adenocarcinoma A549 (0.72 μg/ml) cell lines were closer or lower than that of doxorubicin. Interestingly, low cytotoxicity to normal hepatocyte, AML12 cell line was observed. GA showed anti-angiogenic activity with up to 51.9% inhibition of the growth of blood capillaries on the chorioallantoic membrane of quail embryo. Its also induced apotosis and cell cycle arrest. Ingenuity Pathway Analysis identified several pathways in CCRF-CEM cells and functional group of genes regulated upon GA treatment (*P < 0.05*), the *Cell Cycle: G2/M DNA Damage Checkpoint Regulation* and *ATM Signaling* pathways being amongst the four most involved functional groups.

**Conclusion:**

The overall results of this work provide evidence of the cytotoxic potential of GA and supportive data for its possible use in cancer chemotherapy.

## Background

Malignant diseases are responsible of approximately 13% of all deaths each year in the world. About 12.7 million cancer cases and 7.6 million cancer deaths are estimated to have occurred in 2008; of these, 56% of the cases and 64% of the deaths occurred in the economically developing world [1]. Breast cancer is the most frequently diagnosed cancer and the leading cause of cancer death among females, accounting for 23% of the total cancer cases and 14% of the cancer deaths; Lung cancer is the leading cancer site in males, comprising 17% of the total new cancer cases and 23% of the total cancer deaths [1]. It is reported that cancer is the leading cause of death in the developed world and the second leading cause of death in the developing world [1]. In 2000, leukemia represented about 3% of the almost seven million deaths due to cancer that year, and about 0.35% of all deaths from any cause [2]. Natural products represent a valuable source for the development of novel anticancer drugs. The present study was focused on the cytotoxic potential of a naphtyl butanone, a major component of the leaves of *Guiera senegalensis*, GA. This compound previously showed good antifungal activity against *Cladosporium cucumerinum*[3], strong antiplasmodial activity and high cytotoxicity towards two cancer cell lines: HCT-116 colon carcinoma and THP-1 human acute monocytic leukemia [4]. Interestingly, the complete chemical synthesis of GA was reported [5]. Therefore, we undertook the present work to highlight the anticancer potential of this compound and its possible effects on cell cycle distribution, apoptosis, transcript profiling using leukemia CCRF-CEM cells as a model. The study was also extended to the search of the anti-angiogenic potency of GA.

## Methods

### Chemical for cytotoxicity assay

Doxorubicin (Sigma-Aldrich, Schnelldorf, Germany) was used as a positive (cytotoxic) control. Captopril (Sigma-Aldrich) was used as positive anti-angiogenic control. GA was isolated from the methanol extract from the leaves of *Guiera senegalensis*; The leaves of the plant were collected in Mount Kala (Centre Region of Cameroon, Africa) in May 2008. The plant was identified at the Cameroon National Hebarium (Yaounde) were a voucher specimen was deposited. The air-dried and powdered leaves (1.5 kg) were soaked in 6 L of methanol for 48 h, at room temperature. The methanol extract was concentrated under reduced pressure to give 120 g of a Green-dark residue that constituted the crude extract (GSL). Part of this extract (100 g) was submitted to repeated silica gel 60 (0.04–0.063 mm, 200 g) and thin layer chromatography (TLC) to afforded GA C_18_H_20_O_5_ (light yellow crystals; 48 mg; m/z 316; m.p. 99–101°C) [3,6] (Detailed isolation and general experimental procedures are available as Supporting information).

### Cell lines treatment

A panel of fourteen cancer cell lines including human lymphoblastic CCRF-CEM leukemia cells and their multidrug-resistant subline, CEM/ADR5000, MiaPaCa-2 and Capan*-*1 pancreatic adenocarninoma cells, MCF-7 breast adenocarcinoma cells, SW-680 colon carcinoma cells, 786–0 renal carcinoma cells, U87MG glioblastoma cells, A549 lung adenocarcinoma cells, Caski and HeLa cervical carcinoma cells, Colo-38 skin melanoma cells, as well as AML12 normal hepatocytes were used. Cell lines were obtained from different sources: Prof. Axel Sauerbrey, University of Jena, Jena, Germany (CCRF-CEM, CEM/ADR5000), Dr. Jörg Hoheisel, German Cancer Research Center, Heidelberg, Germany (MiaPaCa-2, Capan-1, MCF-7, SW-680), Tumor Bank, German Cancer Research Center, Heidelberg, Germany (786–0, U87MG, A549, Caski, HeLa, Colo-38), American Type Culture Collection,USA (AML12).

All cell lines were maintained in RPMI 1640 containing 100 units/ml penicillin and 100 μg/ml streptomycin and supplemented with heat-inactivated 10% fetal bovine serum (FBS). All cultured cells were maintained in a humidified incubator at 37°C with 5% CO_2_. Multidrug resistance of CEM/ADR5000 was maintained by applying 5000 ng/ml Doxorubicin every other cell passage. Doxorubicin (Sigma-Aldrich, Schnelldorf, Germany) was used as a positive (cytotoxic) control. The concentration of DMSO was kept at or below 0.1% in all experiments.

#### Resazurin cell growth inhibition assay

Alamar Blue or Resazurin (Promega, Mannheim, Germany) reduction assay [7] was used to assess the cytotoxicity of the studied samples. Doxorubicin was used as positive control. Each assay was done at least three times, with two replicate each. The viability after 48 h was compared based on a comparison with untreated cells. IC_50_ (on cancer cells) or EC_50_ (on AML12 cells) values were the concentration of sample required to inhibit 50% of the cell proliferation and was calculated from a calibration curve by a linear regression [8], using Microsoft Excel (The detailed Resazurin assay is available in Supporting information).

#### Flow cytometry for cell cycle analysis and detection of apoptotic cells

Leukemia CCRF-CEM cells treated with GA or DMSO (solvent control) for 24 to 72 h were fixed with ethanol 95% and washed with cold, phosphate-buffered saline (PBS; Invitrogen) and then resuspended in 150 μl hypotonic fluorochrome solution (50 μg/ml propidium iodide, 10 μg/ml RNAse A in PBS). The cells were incubated in the dark at 4°C overnight before flow-cytometry analysis was performed. The propidium iodide fluorescence of individual nuclei was measured using a FACS-Calibur cytometer (BD Biosciences, Heidelberg, Germany). Data were analyzed with the CellQuess Pro V5.2.1 software (BD Biosciences). For each condition, at least three independent experiments were performed.

#### Caspase-glo 3/7 assay

The influence of GA on caspase 3/7 activity in CCRF-CEM leukemia cells was detected using Caspase-Glo 3/7 Assay kit (Promega). Cells cultured in RPMI were seeded in 96-well plates and treated with the sample (2 × IC_50_; IC_50_; ½ × IC_50_) or DMSO (solvent control). After 24 h treatment, 100 μl of caspase 3/7 reagent were added to each well, mixed and incubated for 1 h at room temperature. Luminescence was measured using well Infinite M2000 Pro™ instrument (Tecan). Caspase 3/7 activity was expressed as percentage of the untreated control.

### Detection of angiogenesis in vivo by cultivation of quail eggs

The quail eggs were purchased from Wachtelzucht Anne Klein, Steinhagen, Germany. The embryos were cultured according to the method described by Wittmann et al. [9]. GA and captopril (as positive control drug) were tested for their anti-angiogenic effects at 20 μg/ml, using chicken chorioallantoic membrane assay (CAM assay) method as described by D’Arcy and Howard [10], with modifications according to Marchesan et al. [11]. The percentage inhibition of vascularization was calculated as previously described [12] (Detailed anti-angiogenic tests are available in Supporting information).

### mRNA-based microarray expression profiling

#### RNA isolation and analysis

Total RNA from CCRF-CEM cells was isolated using RNeasy Kit from Qiagen (Hilden, Germany) according to the manufacture’s instruction. RNA was resuspended/eluted in TE/water. The quality of total RNA was checked by gel analysis using the total RNA Nano chip assay on an Agilent 2100 Bioanalyzer (Agilent Technologies GmbH, Berlin, Germany). Only samples with RNA index values greater than 8.5 were selected for expression profiling. RNA concentrations were determined using the NanoDrop spectrophotometer (NanoDrop Technologies, Wilmington, DE).

#### Probe labeling and illumina sentrix BeadChip array hybridization

Biotin-labeled cRNA samples for hybridization on Illumina Human Sentrix-HT12 BeadChip arrays (Illumina, Inc.) were prepared according to Illumina’s recommended sample labeling procedure based on the modified Eberwine protocol [13] (Detailed tests is available in Supportive information).

#### Scanning and data analysis

Microarray scanning was done using a Beadstation array scanner, setting adjusted to a scaling factor of 1 and PMT settings at 430. Data extraction was done for all beads individually, and outliers are removed when > 2.5 MAD (median absolute deviation). All remaining data points are used for the calculation of the mean average signal for a given probe, and standard deviation for each probe was calculated.

Data analysis was done by normalization of the signals using the quantile normalization algorithm without background subtraction, and differentially regulated genes are defined by calculating the standard deviation differences of a given probe in one-by-one comparisons of samples or groups.

All data is MIAME compliant and the raw data has been deposited in a MIAME compliant database as detailed on the MGED Society website http://www.ebi.ac.uk/arrayexpress/experiments/E-MTAB-731 (Accession number E-MTAB-731).

#### Real-time (RT)-PCR

The same RNA samples used for microarray experiment were also used for RT-PCR experiments. RNA samples extracted from cells treated with GA or DMSO (solvent control) were converted to cDNA by reverse transcriptase (Invitrogen) using random hexamerprimers. The cDNAs were quantified by real-time PCR using the SsoFast EvaGreen PCR Kit (Bio-RAD, München, Germany) and the CFX384^TM^ Real-Time PCR Detection Systems (Bio-RAD), PCR was done by initial incubation at 50°C for 10 min, denaturation at 95°C for 5 min and 40 cycles were performed in two steps for each: denaturation at 95°C for 30 s, annealing at 62°C for 40 s. Melt Curve was performed by cooling from 95°C to 65°C, followed by a gradual increase in temperature (0.5°C/5 s) to 95°C. Expression levels were normalized relative to the transcription level of G6PD. All samples were run in triplicate.

#### Statistical analysis

Statistical analysis of all data was performed using a Student’s *t*-test or Kruskal–Wallis test followed by Dunn’s post-hoc multiple comparison test (Graph-Pad Prism 5.01; GraphPad Software, Inc., CA, USA). *P* < 0.05 denoted significance in all cases.

## Results

### Cytotoxicity

The results of the cytotoxicity of GA on a panel of cancer cell lines are summarized in Table 1. More than 50% inhibition of proliferation after 48 h treatment were obtained for eleven of the twelve studied (91.67%) cancer cell lines, with IC_50_ values below 20 μg/ml. At the concentration of 20 μg/ml, GA did not induced the proliferation of up to 50% for colon SW-680 cell lines as well as for normal AML12 cells (Table 1).

**Table 1:** Cytotoxicity of Guieranone A and Doxorubicin in various cancer cell lines

**Cell lines**	**Sample and IC**_**50**_**values**			
	**Guieranone A**		**Doxorubicin**	
			
	**μg/ml**	**μM**	**μg/ml**	**μM**
CCRF-CEM	**0.73 ± 0.11**	**2.31 ± 0.35**	0.62 ± 0.003	1.14 ± 0.005
CEM/ADR5000	**1.01 ± 0.09 (1.38)**	**3.19 ± 0.28**	>20 (>32.26)	>36.8
MiaPaCa-2	**3.91 ± 0.25**	12.37 ± 0.79	0.95 ± 0.06	1.75 ± 0.11
Capan-1	9.19 ± 2.29	29.08 ± 7.24	4.06 ± 1.18	7.47 ± 2.17
MCF-7	**1.08 ± 0.32**	3.42 ± 0.90	0.59 ± 0.05	1.08 ± 0.09
SW-680	>20	>63.29	0.93 ± 0.09	1.71 ± 0.17
786-0	**3.68 ± 0.54**	11.32 ± 1.70	0.60 ± 0.09	1.10 ± 0.17
U87MG	**2.46 ± 0.02**	7.78 ± 0.05	0.37 ± 0.08	0.68 ± 0.14
A549	**0.72 ± 0.07**	**2.28 ± 0.22**	1.69 ± 0.05	3.11 ± 0.09
Colo-38	**2.43 ± 0.75**	7.69 ± 1.38	0.81 ± 0.06	1.49 ± 0.11
HeLa	**0.51 ± 0.04**	1.61 ± 0.13	0.26 ± 0.02	0.48 ± 0.04
Caski	**1.18 ± 0.11**	3.73 ± 0.35	0.58 ± 0.02	1.07 ± 0.04
AML12 (EC_50_)^*^	>20	>63.29	>20	>36.8

^ac*^EC_50_: effective dose showing 50% inhibition of growth proliferation.

### Anti-angiogenic effect of guieranone A

**Figure 1:**
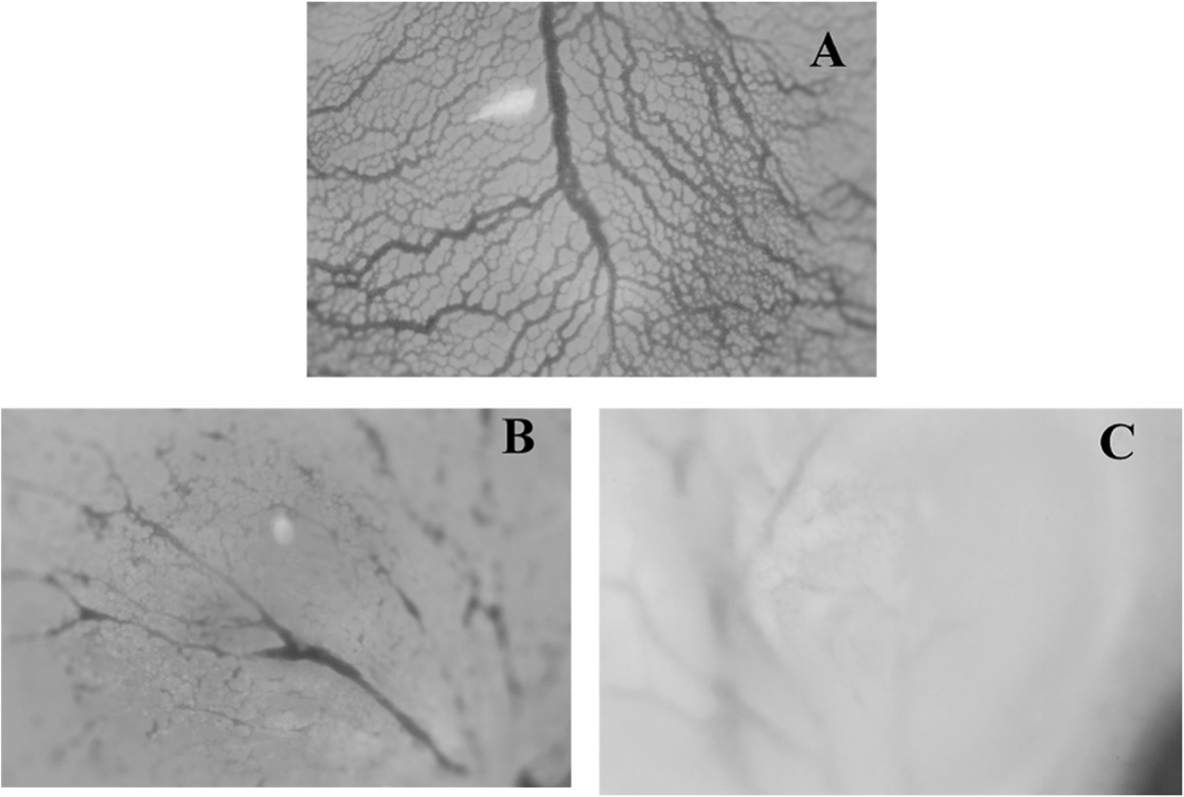
**Effects of Guieranone A (at 20 μg/ml) on the growth of blood capillaries on the chorioallantoic membrane of quail eggs.** (**A**) DMSO (control): Normal growth of blood capillaries on the CAM – no antiangiogenic effect; (**B**): Guieranone A (51.9 ± 1,3% inhibition); (**C**): Captopril (76.46% inhibition ± 3.6%).

In the present study, GA showed 51.9% inhibition of blood capillary growth on the chorioallantoic membrane of quail eggs (Figure 1). However, the anti-angiogenic effect of GA was still lower than that of the reference compound, captopril (76.46% inhibition).

### Cell cycle analysis and apoptosis

**Figure 2:**
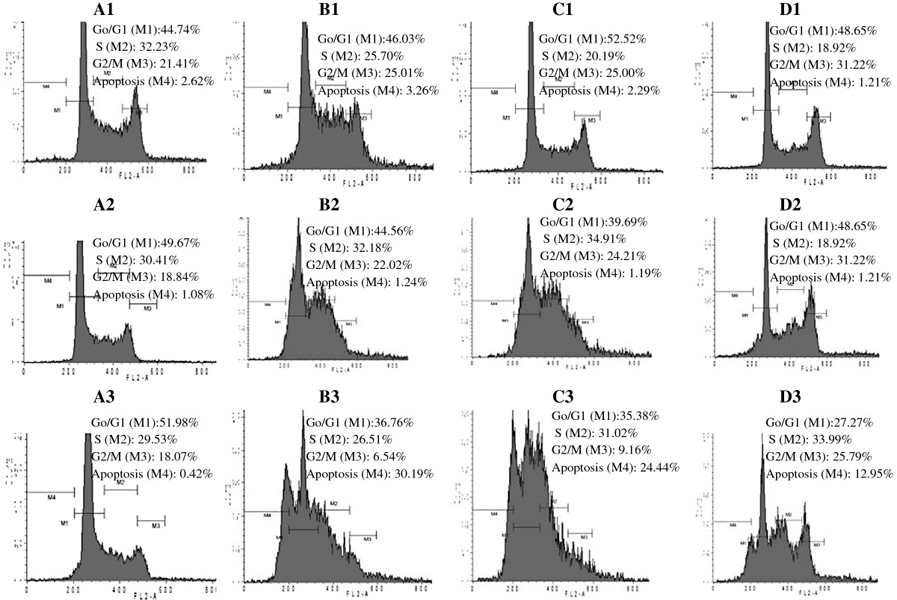
**Cell cycle distribution of CCRF-CEM leukemia cells upon Guieranone A treatment.** A: control; B: Guieranone A at 2 × IC_50_ value; C: Guieranone A at IC_50_ value; D: Guieranone A at 1/2 × IC_50_ value; (1); after 24 h; (2); after 48 h; (3); after 72 h.

The flow cytometry data (Figure 2) indicate that GA did not significantly induce apoptosis in the early phase after treatment of CRRF-CEM cells (24 h), but that the cells progressively underwent apoptosis in a time- and dose-dependent manner (up to 30.19% apoptosis after 72 h at 2 × IC_50_ treatment), and significant reduction of cells in G0/G1 phase and cell cycle arrest betwen S and G2/M phase. This compound also induced low caspase 3/7 activity (Additional file 1: Figure S1) in CCRF-CEM cells after 6 h.

### Microarray analysis and signaling pathway profiling

To identify pathways and possible molecular targets involved in the antitumor effect of GA, CCRF-CEM cells were then treated with this compound and subjected to mRNA-based microarray hybridization. The top 10 up- or down-regulated genes out of 227 genes (see Additional file 1: Table S2) upon GA treatment in CCRF-CEM cells are summarized in Table 2.

**Table 2:** Ten most down- or up-regulated genes in CCRF-CEM cells after treatment guieranone A

**ID**	**Symbol**	**Description**	**Fold Change**
***Up-regulated genes***			
160092	*HSPA6*	heat shock 70 kDa protein 6 (HSP70B)	11.43
290730	*HIST1H2BD*	histone cluster 1, H2bd	8.00
7160239	*FOSB*	FBJ murine osteosarcoma viral oncogene homolog B	5.39
1820592	*HIST2H2AA4*	histone cluster 2, H2aa4	4.45
20129	*CD52*	CD52 molecule	4.16
610451	*HIST2H2AA4*	histone cluster 2, H2aa4	4.07
6510367	*JUN*	jun oncogene	3.86
6660601	*HMOX1*	heme oxygenase (decycling) 1	3.64
1500600	*RAB37*	RAB37, member RAS oncogene family	3.34
6860072	*TRAPPC6A*	trafficking protein particle complex 6A	3.29
***Down-regulated genes***			
2850020	*DHRS2*	dehydrogenase/reductase (SDR family) member 2	−4.17
3420400	*KPNA2*	karyopherin alpha 2 (RAG cohort 1, importin alpha 1)	−4.18
4230196	*KPNA2*	karyopherin alpha 2 (RAG cohort 1, importin alpha 1)	−4.48
460286	*THOC4*	THO complex 4	−4.50
4390315	*HNRNPA1P2*	heterogeneous nuclear ribonucleoprotein A1 pseudogene 2	−4.77
2750719	*DDX21*	DEAD (Asp-Glu-Ala-Asp) box polypeptide 21	−4.91
4890671	*DHRS2*	dehydrogenase/reductase (SDR family) member 2	−5.68
4880646	*ACTBL3*	actin, beta-like 3	−5.70
3940592	*PGAM1*	phosphoglycerate mutase 1 (brain)	−5.76
5270730	*ACTB*	actin, beta	−6.50

*Full list of gene in Supplementary Material.

The Ingenuity Pathway Analysis (version 6.5) identified several pathways (Additional file 1: Tables S3 and S4) and functional group of genes in CCRF-CEM cells which were regulated upon GA treatment (significance value of *P < 0.05*)*.* Amongst the top four functional groups of genes (Table 3), the *Cell Cycle: G2/M DNA Damage Checkpoint Regulation* and *ATM Signaling* pathways (Additional file 1: Figure S1) was directly linked to cancer. CHK1 (Serine/threonine-protein kinase gene; cell cycle control and particularly for entry into mitosis) was found to be down-regulated in these two parthways (Additional file 1: Figure S5). In addition WEE1 (nuclear kinase gene; key regulator of cell cycle progression) (Additional file 1: Table S2) is down-regulated in *Cell Cycle: G2/M DNA Damage Checkpoint Regulation* following GA treatment, giving some explanation on the way of cell death is induced.

**Table 3:** **Functions associated with the top four group of genes (Figures**3, 4**) whose expression was affected by treatment with guieranone A in CCRF-CEM cancer cell lines**

**ID (Network)**	**Score**	**Focus molecules**	Top functions*	**List of all molecules involved**
1	50	28	Molecular transport, Protein trafficking, Cellular assembly and organization	ACP1, ATP1A1, DDX21, DDX3X, DOK2, ERK1/2, GC-GCR dimer, GNL3, Histone h3, Histone h4, HNRNPH1, HNRNPK, Importin alpha, Importin beta, KPNA2, KPNB1, LARP1, LYAR, NCL, NDRG1, NUP62, NUP153, RANBP1, Rnr, RRP1B, RRS1, SLBP, THOC4, TNFRSF8, TNPO1, TPX2, TSC22D3, TSR1, WDR12, YBX1
2	50	28	Drug metabolism, endocrine System development and function, lipid metabolism	ACTB, Actin, ACTL6A, Akt, alcohol group acceptor phosphotransferase, CS, FASN, HMGA1, HNRNPL, HSP90AB1, IARS, IKK (complex), IPO4, KEAP1, Lamin b, MAP3K8, MCM3, MCM4, MORF4L2, MYCN, ORC6L, PDCD6IP, PSMD6, PTGES3 (includes EG:10728), RNF4, RPL6, RPL7A (includes EG:6130), RPS24 (includes EG:6229), RUVBL1, SGK1, SLC38A2, SMC3, Thyroid hormone receptor, TIP60, TUBB
3	47	27	Cell death, cell-to-cell signaling and interaction, cellular function and maintenance	14-3-3, ACAT2, AHCY, APIP, ARF3, CD3, CD52, CTPS, FAM83D, FOSB, FOXO3, GADD45B, HBP1, HNRNPAB, HSPA6, IL10RB, Immunoglobulin, KIF23, LMNB1, Mapk, Mek, MVP, PDGF BB, Pld, PPHLN1, PPP1R15A, PRDX3, PRKCB, RBCK1, RGS2, TCR, UTP14A, VIM, YWHAE, YWHAG
4	45	26	Cell cycle, cancer, gastrointestinal disease	ADAR, APC, CCNA2, CDK1, CDKN3, CHEK1, CNOT7, Cyclin A, DHFR, E2f, FBXO5, FEN1, H2AFY, ID2, IFI16, KIAA0101, MAD2L1, MAP2K1/2, methenyltetrahydrofolate cyclohydrolase, methylenetetrahydrofolate dehydrogenase (NADP), Mre11, MTHFD1, MTHFD2, MTHFD1L, NBN, NUSAP1, PI3K (complex), PRIM1, Rb, RFC4, TFDP1, TINF2, TYMS, WEE1, XRCC5

*List of all function in Supplementary Material S8. The score is the rounded negative base-ten logarithm of a p-value that measures the likelihood of genes in the network or functional cluster occuring by chance. A score of 50 means that a cetrain network or cluster has the approximately likelihood of 10^-50^ of occuring by random chance.

**Figure 3:**
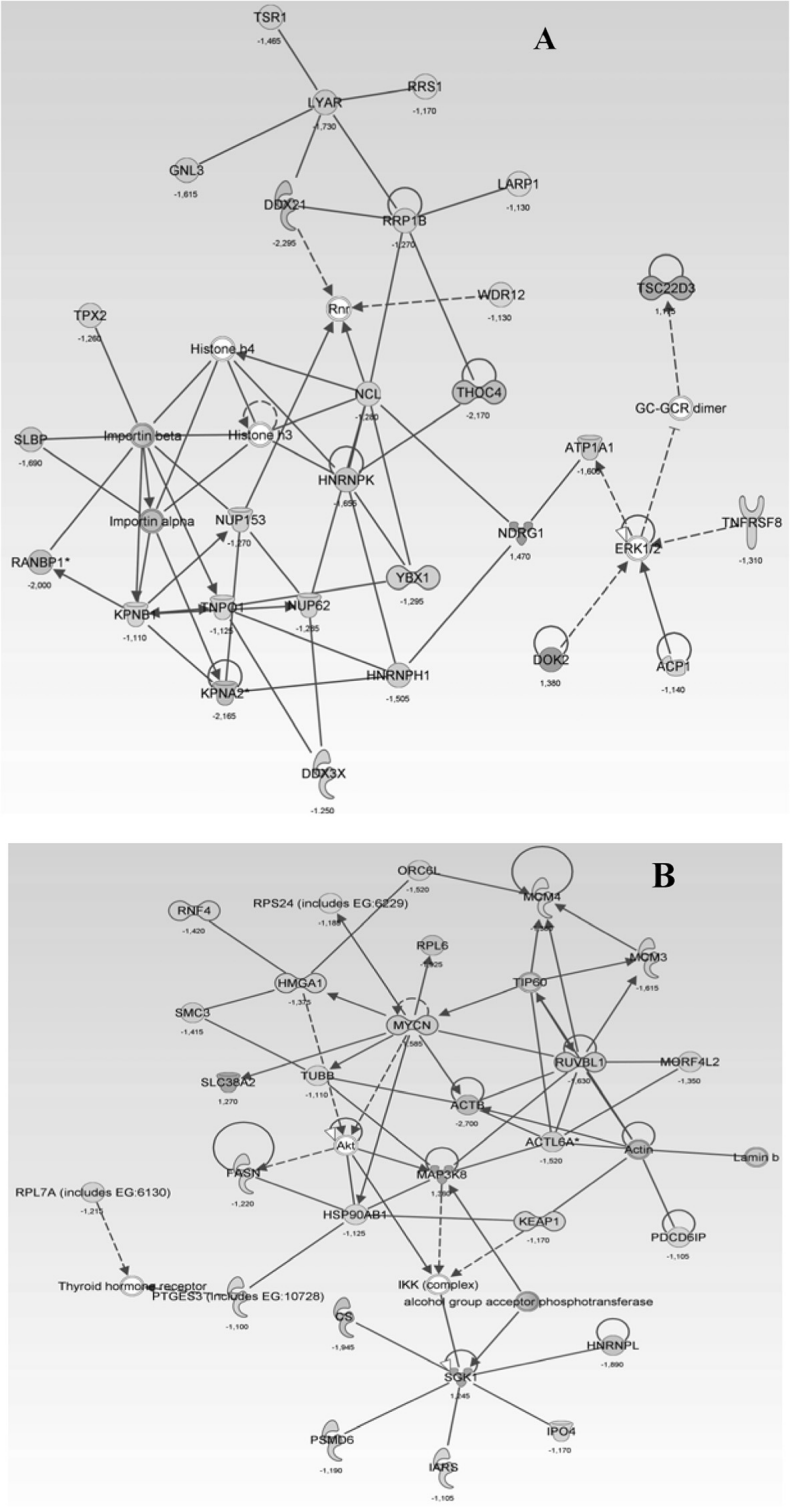
**Network of differentially regulated genes upon Guieranone A treatment.** (**A**) Molecular transport, protein trafficking, cellular assembly and organization, (**B**) Drug metabolism, endocrine system development and function, lipid metabolism. (+): upregulted; (−): downregulated.

**Figure 4:**
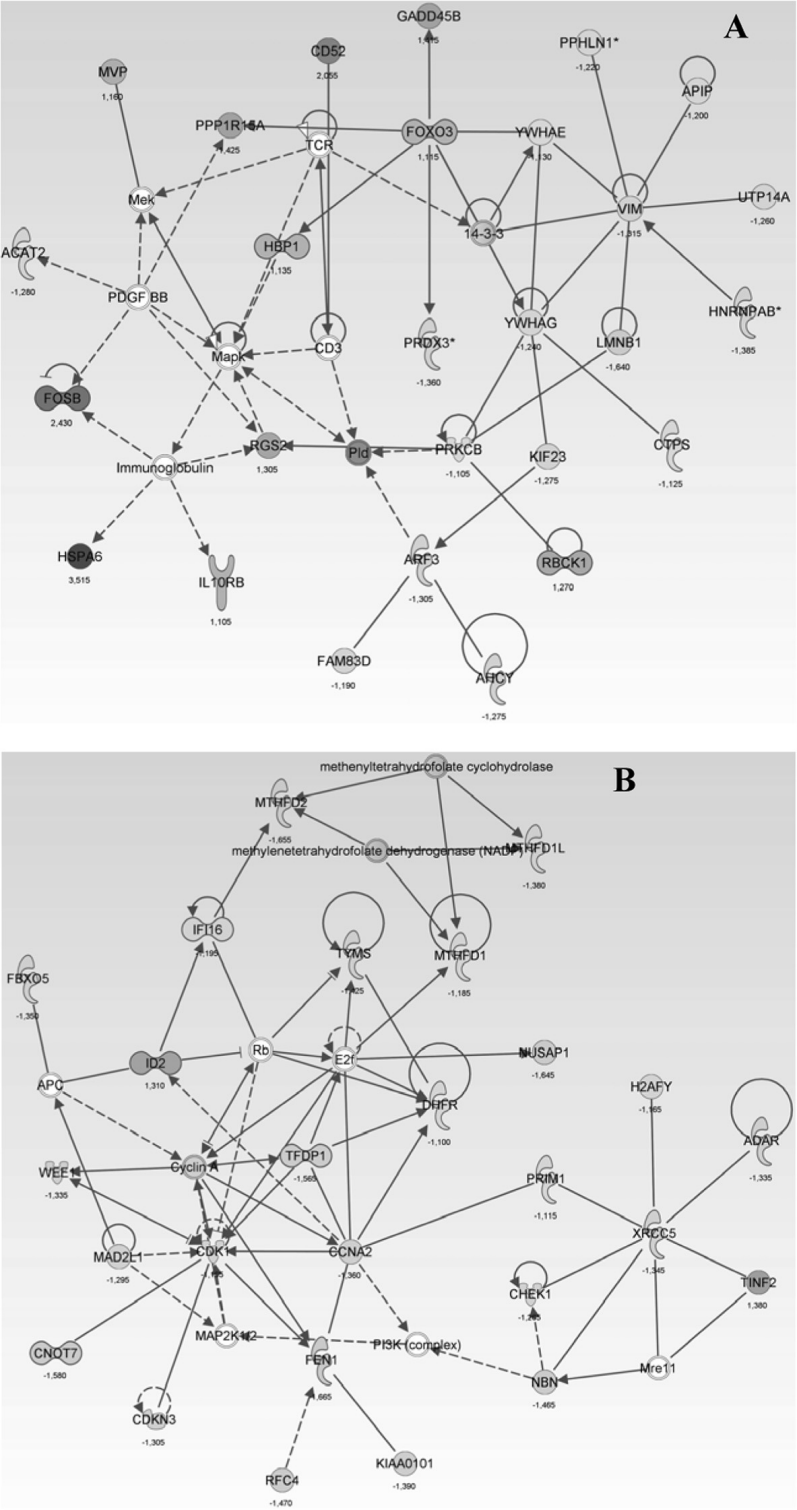
**Network of genes of involved (upon guieranone A treatment) in Cell death, cell-to-cell signaling and interaction, cellular function and maintenance (A), and Cell cycle, cancer, gastrointestinal disease (B).** (+): upregulted; (−): downregulated.

The networks representing the four most important functional group of genes affected by GA treatment are illustrated in Figures 3 and 4. The two most up-regulated genes are *HSPA6* (heat shock 70 kDa protein 6) and *HIST1H2BD* (histone cluster 1, H2bd) (Table 2, Figure 4A). Further genes of interest which are up-regulated are *FOSB* and *JUN*, *HIST1H2BD, HIST2H2AC, HIST2H2AA4,* CD52 (Table 3). Important down-regulated genes found upon GA treatment were *ACTB* and *ACTBL3*, *PGAM1*, *LOC728188*, *DHRS2*, *KPNA2*, *THOC4, RAB37* and *TRAPPC6A*, *HNRNPK*, *LYAR* and *YBX1, LYAR, YBX1, MYCN*, *RUVBL1* (Figure 3 and 4).

### Real time RT-PCR

To verify the microarray data with a second independent method, RT-PCR was exemplarily performed for some differentially regulated genes (i.e. *ACTB, GADD45, HSP90AB1, LYAR* as well as for G6PD as reference). All results were in accordance with the microarray results (see Additional file 1: Figure S6).

## Discussion

### Cytotoxicity

In the US NCI plant screening program, a compound is generally considered to have *in vitro* cytotoxic activity, if the IC_50_ value following incubation between 48 and 72 h, is less than 4 μg/ml or 10 μM [14]. In the present work, the IC_50_ values below 4 μg/ml were recorded on 11/14 (75.57%) of the studied cancer cells, clearly highlighting the good cytotoxicity potential of GA. In addition, values obtained on CCRF-CEM (IC_50_ of 0.73 μg/ml) and its multidrug-resistant subline, CEM/ADR5000 (1.01 μg/ml), A549 (0.72 μg/ml) were closer or lower than that of doxorubicin, clearly confirming this statement. Besides, GA was active on multidrug-resistant CEM/ADR5000 cells, suggesting that this compound might be useful for cancer therapy, including tumor cells resistant to some of the commonly used anticancer drugs. As the liver is the main organ involved in drug metabolism, AML12 normal hepatocytes were used to evaluate the cytotoxicity of GA in normal cells. Interestingly, the EC_50_ > 20 μg/ml obtained was higher than the values obtained against most of the cancer cells (Table 1). The overall results of the cytotoxicity assay indicated that GA might be a promising candidate as new anticancer drug. Therefore, further studies were conducted to investigate the possible mode of action of this compound.

### Anti-angiogenic effect of guieranone A

Angiogenesis, the formation of new blood vessels within a tumor (or many other tissue types) has become a target of pharmacological research as well as industrial drug discovery [15]. Compounds with anti-angiogenic properties are of importance in the treatment and prevention of malignancies as well as other chronic diseases [16]. Though GA showed less antiangiogenic activity than captopril, the result obtained indicates that, in addition to direct cytotoxicity on cancer cells, this compound could also inhibit the proliferation of blood vessels in vivo, presumably with negative effect on tumor progression.

### Cell cycle analysis and apoptosis

The low induction of apoptosis at the early stage of CCRF-CEM cells proliferation is in consistence with the caspase 3/7 activity study (Additional file 1: Figure S1), as low activation of these apoptosis effectors was noted upon 6 h. However, higher apoptosis rates were finally obtained after 72 h with all tested concentrations, showing that apoptotic pathways are involved in the mechanism of GA-induced cell death in CCRF-CEM leukemia cells. Therefore, in vivo bioavailability and bioactivity studies are also to be carried out with this compound, as GA showed promising *in vitro* activities but induces apoptosis upon a long time period (72 h).

### Microarray analysis and signaling pathway profiling

The cell cycle arrest in CCRF-CEM cells was early detected after 24 h and the down regulation of important genes such as CHK1 and WEE1, as reported above might plays a considerable role. This allegation is strengthened by the fact that one of the key players in cell cycle regulation such as GADD45 is also up-regulated following GA treatment (Additional file 1: Figure S1).

*HSPA6* (heat shock 70 kDa protein 6) and *HIST1H2BD* (histone cluster 1, H2bd) genes were found to be most up-regulated upon GA treatment. In cooperation with other chaperones, Hsp70s stabilize preexistent proteins against aggregation and mediate the folding of newly translated polypeptides in the cytosol as well as within organelles. They bind extended peptide segments with a net hydrophobic character exposed by polypeptides during translation and membrane translocation, or following stress-induced damage (http://www.uniprot.org). Interestingly, 70 kDa heat shock protein protects cells from ischemia and its expression is increased in consequence to hypoglycemia [17,18]. In addition, closely connected genes to *HSPA6, HSPA1A* and *DNAJB2*, were found to be up-regulated in our anaylses. Whereas *HSPA1A* is coding for heat shock 70 kDa protein 1A/1B, *DNAJB2* has as protein product cochaperon Hsp40 (DNAJ in bacteria) which is essential for Hsp70 function [19]. Interesting genes which are up-regulated included *FOSB* and *JUN*. A combined up-regulation of these genes with Hsp70 mRNA was earlier observed in as stress response to high acceleration [20]. This finding also confirmed the assumption that GA might cause hypoxic stress. Through high acceleration an induction of ischemia can be observed [20]. Furthmore, dimerization of protein products of FOSB (a FOS family member) and JUN constitute the transcription factor AP-1 which is activated by oxidative stress [21]. Surprisingly, a cofactor of JUN-activated transciption, *DDX21*, belonged to one of the most down-regulated genes in our microarray analysis [22]. This findings give reason to speculate that GA might mimic under-supply of oxygen or glucose and, therefore, leads to apoptosis (see also Figure 2). Very interesting in the contest of the latter assumption was that GA inhibited angiogenesis (Figure 1). It would be worthwhile to investigate, whether the vascular endothelial growth factor (VEGF), its secretion or even the VEGF receptor are inhibited by GA leading to interrupted VEGF signaling, as normally VEGF is activated under hypoxic conditions [23]. Thereby, the fact that we did not observed any significant change in *VEGF* mRNA expression (See Additional file 1: Table S3) does not mean that VEGF signaling was not targeted by GA. VEGF protein expression can independently vary from corresponding mRNA amounts in the cell [24].

Certain histone mRNAs were also up-regulated after treatment with GA *HIST1H2BD, HIST2H2AC, HIST2H2AA4* (Table 3)]. This observation is surprising, as levels of histone mRNA usually increase during S-phase, but decrease back to baseline level between the S-phase and mitosis [25]. This finding was also confirmed by our FACS analyses showing that cells treated with 1 × IC_50_ of GA for 72 h were more often arrested in the S- or G2/M phases than untreated control cells (see also Figure 2.C2 and D2).

CD52 is a membrane protein which is discussed as target molecule for leukemia therapy [26]. Alemtuzumab is an antibody directly targeting CD52 and already approved for clinical use [27]. In our experiments, the mRNA of this protein was strongly up-regulated. A combined treatment of GA with Ametuzumab may possibly boost the apoptotic effect of both drugs, as GA might sensitize cells to Alemtuzumab by inducing CD52 expression and activating CD52 downstream mechanisms. This speculations merits further experimentation in the future.

*ACTB* and *ACTBL3* belonged to the most down-regulated genes. Beta-actin mRNA levels are known to be disturbed after ischemia [28], which is in line with our assumption that GA may mimic hypoxia. Another gene fitting to our hypothesis is *PGAM1*, which codes for phosphoglycerate mutase in glycolysis. Another gene coding for a protein similar to phosphoglycerate mutase processed protein was also down-regulated by GA, *LOC728188*. Down-regulation of glycolysis key molecules accompanied by hypoxic stress may destroy the entire energy production apperture ultimately leading to cell death. The mis-regulation in glyco-related mechanisms was also indicated by down-regulation of *DHRS2*, whose encoded protein preferentially binds to glucose and related sugars [29].

*KPNA2* codes for importin alpha. This protein is a key player in the nuclear transport of macromolecules [30]. Moreover, *HNRNPA1P2* is rarely investigated yet, but seems to be involved to mRNA transport from the nucleus to cytosol (according to UniProt database, http://www.uniprot.org). *THOC4* encoding a more investigated mRNA transporter molecule was also significantly down-regulated. The THOC4 protein is part of the TREX complex, which specifically associates with spliced mRNA [31]. THOC4 is especially involved in nuclear export of Hsp70 transcripts [32].

Interestingly, *RAB37* and *TRAPPC6A* encode also two proteins which are also involved to transport mechanisms (http://www.uniprot.org). They were also mis-regulated in their transcriptional activity after GA treatment. In summary, it seems that transport mechanisms are de-regulated as consequence of treatment with this compound.

Recapitulating, GA seems to cause hypoxia and hypoglycemia as several genes of these functions were affected. Furthermore, a considerable de-regulation of several protein- and mRNA-transporter genes was observed. The latter findings was also confirmed by Ingenuity Pathway Analyses of the microarray data pointing to “Molecular Transport” and “Protein Trafficking” with highest score (Table 3; Figure 3). In contrast, no molecules belonging to the other hits of Table 3 were found to be extremely up- or down-regulated.

In the signaling networks of Figures 3 and 4, more key players become apparent which do not belong to most mis-regulated genes in our investigation: *HNRNPKLYAR* and *YBX1*. According to UniProt database, HNRNPK is involved in RNA processing and splicing. However, it is interesting from our point of view that it seems to be important for enhanced proliferation, as increased levels of *HNRNPK* mRNA correlates with increased proliferative activity [33]. *LYAR* is a gene involved in development and cell growth regulation and especially for leukemia cells, as mRNA levels are increased, but LYAR mRNA is not or only less expressed in cells of different healthy tissues, *e.g.* thymus, bone marrow, liver, heart, brain, kidney or spleen [34]. *YBX1* encodes for a protein, YB-1, which is involved in many mechanisms: proliferation, mRNA processing, DNA repair, transcription, splicing and drug resistence [35]. The relation of YB-1 to drug resistance is due to its activation of the multidrug resistance gene, *MDR1*[36]. In addition, *YBX1* expression is also important for deregulation of the oncogene, *MYCN*[37] (Figure 3B).

MYCN is a prominent transcription factor important for tumorigenesis and chemotherapy. *MYCN* mRNA was found to be over-expressed in human tumor biopsies [38]. Directly correlated to this finding is the down-regulation of *RUVBL1* mRNA (Figure 3B) encoding a protein which binds to MYC [39]. It is essential for cell proliferation. In addition, the RUVBL1 protein is associated with the activation of NuA4 histone acetyltransferase complex, which is associated to the de-regulated histone mRNAs mentioned above [40]. Finally, *SGK1* up-regulation is very interesting, as the encoded protein might counteract the cytotoxic activity of GA and activate cell survival processes. It deserves more detailed investigations in the future to elucidate the connection between KPNA2 and SGK1, as recognition by importin alpha of Sgk (protein of SGK1 gene) might be necessary for nuclear import of activated SGK1 [41].

Furthermore, there is another connection between *SGK1* (Figure 3B) and *FOXO3A* (Figure 4A). FOXO3A is a pro-apoptotic protein which is regulated by SGK1 [42]. FOXO3A has also an important role as transcription factor in oxidative stress reponse and cell protection [43]. Its role in cellular response to GA needs further investigations, because the activity of FOXO3A protein is controlled by phosphorylation, which was not measured in our mRNA expression profiling approach. Another prominent member involved in cell death mechanisms is *YWHAG*, which codes for 14-3-3 gamma protein [44]. 14-3-3 proteins are directly involved in apoptosis and cell survival regulation by inhibiting BAD and FOXO3A [45]. In summary, the signaling network shown in (Figure 3B) depicts genes related to cell survival.

The overall results of the present investigation strengthened the cytotoxic potency, the effect on cell cycle distribution, apoptosis, angiogenesis of Guieranone A, and consequently give important information for the future investigation, that could lead to the potential use of this compound in cancer therapy.

## Competing interests

No potential conflicts of interest were disclosed.

## Author’s contributions

Conceived and designed the experiments: VK, TEi, BW, BK and TE. Analyzed the data: VK, and TE. performed the experiments: VK, TEi, BK. Wrote the paper: VK, TEi and TE. All authors read and approved the final manuscript.

## Supplementary Material

Additional file 1 **Figure S1.** Custumized regulated pathways affected by guieranone A treatment in CCRF-CEM cells (A). (B) Cell cycle: G2/M DNA damage checkpoint regulation; (C): ATM signaling. **Table S2.** Complete list of Signaling pathways with corresponding genes affected by treatment of CCRF-CEM cells with guieranone A. **Table S3.** Enzymatic activity of caspase 3/7 after 6 h treatment of CCRF-CEM cells. The activity of caspase 3/7 is expressed as percentage % relative to untreated cells. **Figure S4.** Top 10 signaling pathways affected by guieranone A treatment in CCRF-CEM cells. The evaluation of differentially expressed genes was performed using the Ingenuity Pathway Analysis software. (List of all pathways in supplemental data 8). **Figure S5.** Genes down- or up-regulated in CCRF-CEM cells after treatment guieranone A. **Figure S6.** Results of real-time reverse transcriptase PCR analysis. CCRF-CEM cells were treated with IC_50_ concentration of guieranone for 24 h, Transcriptional changes are expressed relative to G6PD. The mean value ± SEM of three independent experiments is shown. **Table S7.** Functions associated with the networks for genes whose expression was affected by treatment with guieranone A. Click here for file
