# Supplementary material for: Cytotoxicity, anti-angiogenic, apoptotic effects and transcript profiling of a naturally occurring naphthyl butenone, guieranone A
Source: Cell Div. 2012 Jun 20;7:16. doi: 10.1186/1747-1028-7-16 (PMC3782753; doi:10.1186/1747-1028-7-16)
Supplement: Additional file 1 — Figure S1. Custumized regulated pathways affected by guieranone A treatment in CCRF-CEM cells (A). (B) Cell cycle: G2/M DNA damage checkpoint regulation; (C): ATM signaling. Table S2. Complete list of Signaling pathways with corresponding genes affected by treatment of CCRF-CEM cells with guieranone A. Table S3. Enzymatic activity of caspase 3/7 after 6 h treatment of CCRF-CEM cells. The activity of caspase 3/7 is expressed as percentage % relative to untreated cells. Figure S4. Top 10 signaling pathways affected by guieranone A treatment in CCRF-CEM cells. The evaluation of differentially expressed genes was performed using the Ingenuity Pathway Analysis software. (List of all pathways in supplemental data 8). Figure S5. Genes down- or up-regulated in CCRF-CEM cells after treatment guieranone A. Figure S6. Results of real-time reverse transcriptase PCR analysis. CCRF-CEM cells were treated with IC50 concentration of guieranone for 24 h, Transcriptional changes are expressed relative to G6PD. The mean value ± SEM of three independent experiments is shown. Table S7. Functions associated with the networks for genes whose expression was affected by treatment with guieranone A. [file 1747-1028-7-16-S1.doc]

**Cytotoxicity, anti-angiogenic, apoptotic effects and transcript profiling of a naturally occurring naphthyl butenone, Guieranone A**

Victor Kuete^1,2*^, Tolga Eichhorn^2^, Benjamin Wiench^2^, Benjamin Krusche^2^, Thomas Efferth^2**^

*^1^Department of Biochemistry, Faculty of science, University of Dschang, Cameroon,*

*^2^Department of Pharmaceutical Biology, Institute of Pharmacy and Biochemistry, University of Mainz, Staudinger Weg 5, 55128 Mainz, Germany.*

*****Corresponding author:**

** Tel: (+237) 77 35 59 27/(+237) 75468927; Fax: (+237) 222 60 18; E-mail:* [*kuetevictor@yahoo.fr*](mailto:kuetevictor@yahoo.fr) *(Dr Victor Kuete)*

***Tel: (+49) 6131-3925751; Fax: (+49) 49-6131-3923752; E-mail:* [*efferth@uni-mainz.de*](mailto:efferth@uni-mainz.de)*; 55128 Mainz, Germany (Prof. Dr. Thomas Efferth)*

**[**Supporting information **(S): See Main Document]**

***Methods***

***Isolation and identification of GA.***

The air-dried and powdered leaves (1.5 kg) were soaked in 6 L of methanol for 48 h, at room temperature. The methanol extract was concentrated under reduced pressure to give 120 g of a Green-dark residue that constituted the crude extract (GSL). Part of this extract (100 g) was submitted to silica gel 60 (0.04-0.063 mm, 200 g) vacuum flash chromatography using as eluent, hexane (Hex, 8750 ml), hexane-ethyl acetate (Hex-EtOAc) 75:25 (5000 ml), Hex-EtOAc 50:50 (7000 ml), (Hex-EtOAc) 25:75 (4500 ml), EtOAc (3000 ml) and methanol (MeOH, 2000 ml). One hundred twenty one (121) sub-fractions of 250 ml each were collected and pooled on the basis of their thin layer chromatography (TLC) profile in four main fractions (frs), GSL1 (subfrs 1-13; 15 g), GSL2 (subfrs 14-42; 28 g), GSL3 (subfrs43-94; 22 g) and GSL4 (95-121; 17 g). GSL2 was then submitted to silica gel 60 column chromatography using hexane and a mixture of Hex-EtOAc of increasing polarity as eluent. Ninety seven (97) sub-fractions of 100 ml each were collected and sub-frs 22-28 obtained with Hex-EtOAc 80:20 afforded GA C_18_H_20_O_5_ ( light yellow crystals; 48 mg; m/z 316; m.p. 99-101 ˚C) [3, 6].

***General procedure:*** Aluminum sheet pre-coated with silica gel 60 *F*254 nm (Merck) was used for thin layer chromatography. The spots were visualized using both ultraviolet light (254 and 366 nm) and 50% H_2_SO_4_ spray reagent. NMR spectra were recorded on a Bruker Avance 300 at 300MHz (1H) and 75MHz and Bruker Avance 600 at 600MHz (^1^H) and 150MHz (^13^C), with the residual solvent peaks as internal references. The melting point (m.p.) was determined using a Kofler microhot stage apparatus. Mass spectra were recorded with API QSTAR pulsar mass spectrometer. The structures of GA was confirmed by comparing with reference data from available literature.

***Resazurin cell growth inhibition assay:*** Alamar Blue or Resazurin (Promega, Mannheim, Germany) reduction assay [7] was used to assess the cytotoxicity of the studied samples. The assay tests cellular viability and mitochondrial function. Briefly, adherent cells were grown in tissue culture flasks, and then harvested by treating the flasks with 0.025% trypsin and 0.25 mM EDTA for 5 min. Once detached, cells were washed, counted and an aliquot (5×10^3^ ells) was placed in each well of a 96-well cell culture plate in a total volume of 100 µl. Cells were allowed to attach overnight and then treated with samples. After 48 h, 20 µl resazurin 0.01% w/v solution was added to each well and the plates were incubated at 37 ˚C for 1-2 h. Fluorescence was measured on an automated 96-well Infinite M2000 Pro™ plate reader (Tecan, Crailsheim, Germany) using an excitation wavelength of 544 nm and an emission wavelength of 590 nm. For leukemia cells, aliquot of 5×10^4^ cells/ml (obtained from overnight suspension) were seeded in 96-well plates, and extracts were added immediately. After 24 h incubation, plates were treated with resazurin solution as mentioned above. Doxorubicin was used as positive control. Each assay was done at least three times, with two replicate each. The viability was compared based on a comparison with untreated cells. IC_50_ (on cancer cells) or EC_50_ (on AML12 cells) values were the concentration of sample required to inhibit 50% of the cell proliferation and was calculated from a calibration curve by a linear regression [8], using Microsoft Excel.

***Detection of angiogenesis in vivo by cultivation of quail eggs***

The quail eggs were purchased from Wachtelzucht Anne Klein, Steinhagen, Germany. The embryos were cultured according to the method described by Wittmann et al. [9]. Briefly, fertilized quail eggs were incubated for 70 h at 38 °C and 80% relative humidity. After 70 h of incubation the eggs were opened. For this purpose the eggs were placed in a vertical position to guarantee that the embryo floats in the upper part of the egg. Afterwards, a hole was cut into the top of the egg and the complete content of the egg was transferred into a Petri dish. By using this method, it could be guaranteed that the albumin gets first into the Petri dish followed by the yolk with the embryo on top without exposing the embryo to shock-forces which could damage the vitelline membrane.

Guieranone A and captopril (as positive control drug) were tested for their anti-angiogenic effects using the method of chicken chorioallantoic membrane assay (CAM assay) described by D’Arcy and Howard [10] with modifications according to Marchesan et al. [11]. Briefly, the explanted embryo was placed in an incubator for 2 h at 38 °C to acclimatize to the new ambience. Subsequently, the test substance was placed on the chorioallantoic membrane (CAM). Therefore, 2 % agarose solution was prepared and mixed 1:10 with GA prior diluted in DMSO 0.1% final concentration. The final concentration of the substance was 20 µg/ml. Pellets with 0.1% DMSO served as control. The agarose-pellets were then placed on the chorioallantoic membrane after they cooled down to room temperature. The Petri dishes with the quail embryos were placed in the incubator again and incubated at 38 °C and 80% relative humidity for 24 h before documenting the effect of the applied substance.

Imaging of the vascularized quail eggs was performed using a digital camera with 3x-magnification objective (Canon eos 500 with a canon mp-e 65 2.8 macro objective). For illumination a mercury-arc-lamp was used which provided a high fraction of blue and UV-light to obtain good contrast values between yolk and vessels. The pictured image section had a size of 5×5 mm. Following image acquisition, quantitative analysis was performed using a software routine which was written in the ImageJ-macro language, and the total small vessels number (or area) was then determined by the system. The percentage inhibition of vascularization was calculated as previously described [12].

***Probe labeling and Illumina Sentrix BeadChip array hybridization:*** Biotin-labeled cRNA samples for hybridization on Illumina Human Sentrix-HT12 BeadChip arrays (Illumina, Inc.) were prepared according to Illumina's recommended sample labeling procedure based on the modified Eberwine protocol [13]. In brief, 250 ng total RNA was used for complementary DNA (cDNA) synthesis, followed by an amplification/labeling step (*in vitro* transcription) to synthesize biotin-labeled cRNA according to the MessageAmp II aRNA Amplification kit (Ambion, Inc., Austin, TX). Biotin-16-UTP was purchased from Roche Applied Science, Penzberg, Germany. The cRNA was column purified according to TotalPrep RNA Amplification Kit, and eluted in 60 µl of water. Quality of cRNA was controlled using the RNA Nano Chip Assay on an Agilent 2100 Bioanalyzer and spectrophotometrically quantified (NanoDrop).

Hybridization wais performed at 58°C, in GEX-HCB buffer (Illumina) at a concentration of 100 ng cRNA/µl, unsealed in a wet chamber for 20h. Spike-in controls for low, medium and highly abundant RNAs were added, as well as mismatch control and biotinylation control oligonucleotides. Microarrays were washed once in High Temp Wash buffer (Illumina) at 55°C and then twice in E1BC buffer (Illumina) at room temperature for 5 minutes (in between washed with ethanol at room temperature). After blocking for 5 min in 4 ml of 1% (wt/vol) Blocker Casein in phosphate buffered saline Hammarsten grade (Pierce Biotechnology, Inc., Rockford, IL), array signals are developed by a 10 min incubation in 2 ml of 1 µg/ml Cy3-streptavidin (Amersham Biosciences, Buckinghamshire, UK) solution and 1% blocking solution. After a final wash in E1BC, the arrays are dried and scanned.

**S1.** Custumized regulated pathways affected by guieranone A treatment in CCRF-CEM cells (A). (B) Cell cycle: G2/M DNA damage checkpoint regulation; (C): ATM signaling.


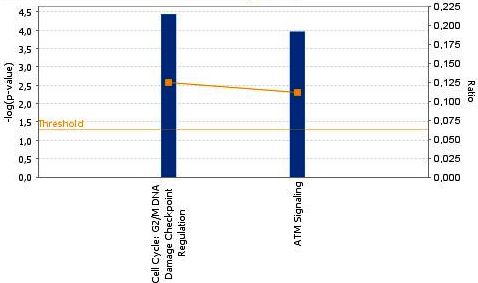

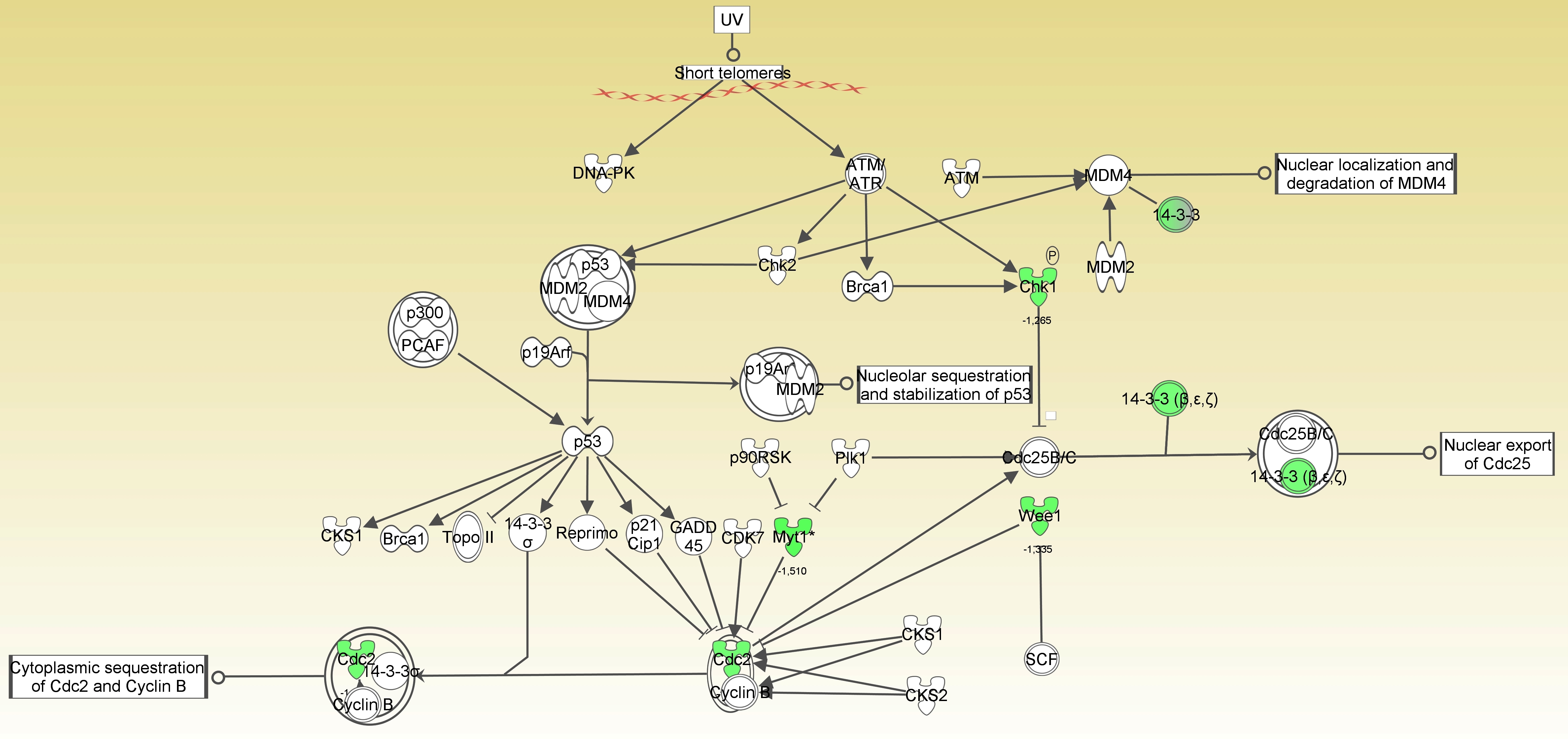


**(B)**


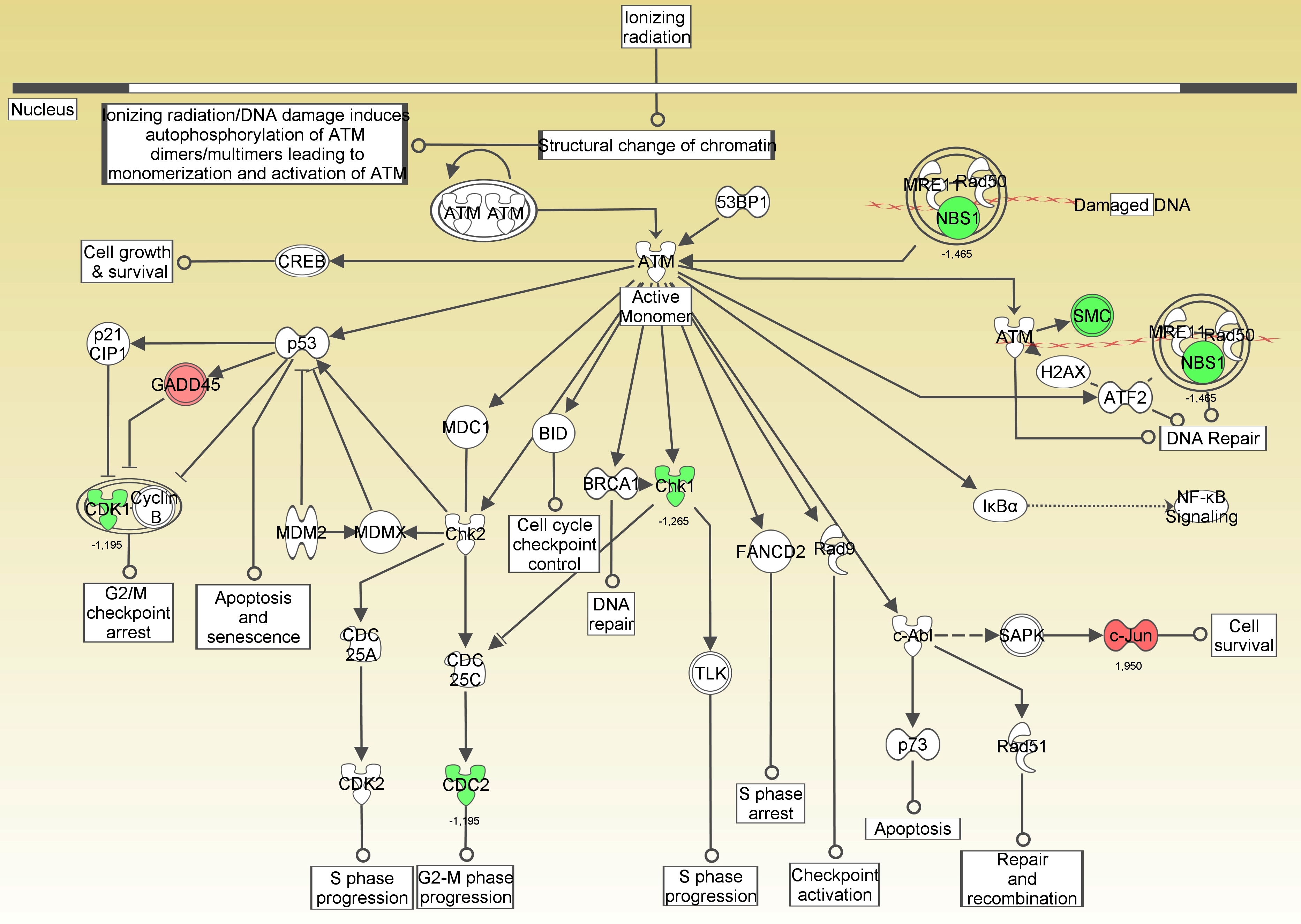


**(A)**

**(C)**

**Table S2. Genes down- or up-regulated in CCRF-CEM cells after treatment guieranone A.**

| **order** | **ID** | **Symbol** | **Description** | **Fold**  **Change** |
| --- | --- | --- | --- | --- |
|  | 160092 | HSPA6 | heat shock 70kDa protein 6 (HSP70B) | 11.43 |
|  | 290730 | HIST1H2BD | histone cluster 1, H2bd | 8.00 |
|  | 7160239 | FOSB | FBJ murine osteosarcoma viral oncogene homolog B | 5.39 |
|  | 1820592 | HIST2H2AA4 | histone cluster 2, H2aa4 | 4.45 |
|  | 20129 | CD52 | CD52 molecule | 4.16 |
|  | 610451 | HIST2H2AA4 | histone cluster 2, H2aa4 | 4.07 |
|  | 6510367 | JUN | jun oncogene | 3.86 |
|  | 6660601 | HMOX1 | heme oxygenase (decycling) 1 | 3.64 |
|  | 1500600 | RAB37 | RAB37, member RAS oncogene family | 3.34 |
|  | 6860072 | TRAPPC6A | trafficking protein particle complex 6A | 3.29 |
|  | 130750 | YPEL3 | yippee-like 3 (Drosophila) | 3.20 |
|  | 2680097 | YPEL5 | yippee-like 5 (Drosophila) | 3.08 |
|  | 5340279 | NA | NA | 3.08 |
|  | 6100022 | HIST2H2AC | histone cluster 2, H2ac | 3.06 |
|  | 3990379 | ITGB7 | integrin, beta 7 | 3.06 |
|  | 2970044 | ABTB1 | ankyrin repeat and BTB (POZ) domain containing 1 | 2.99 |
|  | 4760433 | C16orf7 | chromosome 16 open reading frame 7 | 2.91 |
|  | 1440452 | N4BP2L2 | NEDD4 binding protein 2-like 2 | 2.87 |
|  | 3390128 | TPM2 | tropomyosin 2 (beta) | 2.81 |
|  | 3460376 | RTN2 | reticulon 2 | 2.80 |
|  | 130519 | STAT2 | signal transducer and activator of transcription 2, 113kDa | 2.80 |
|  | 1230070 | NDRG1 | N-myc downstream regulated 1 | 2.77 |
|  | 1510681 | NBPF10 | neuroblastoma breakpoint family, member 10 | 2.73 |
|  | 130156 | PDE1B | phosphodiesterase 1B, calmodulin-dependent | 2.72 |
|  | 7210035 | NA | NA | 2.72 |
|  | 5960086 | RNU11 | RNA, U11 small nuclear | 2.69 |
|  | 1340600 | PPP1R15A | protein phosphatase 1, regulatory (inhibitor) subunit 15A | 2.69 |
|  | 1170605 | LPAR2 | lysophosphatidic acid receptor 2 | 2.69 |
|  | 7400053 | SLC22A18 | solute carrier family 22, member 18 | 2.68 |
|  | 4920110 | GADD45B | growth arrest and DNA-damage-inducible, beta | 2.67 |
|  | 6380717 | HSPA1A | heat shock 70kDa protein 1A | 2.67 |
|  | 5810678 | RENBP | renin binding protein | 2.63 |
|  | 2760452 | RPS29 | ribosomal protein S29 | 2.62 |
|  | 1740471 | TINF2 | TERF1 (TRF1)-interacting nuclear factor 2 | 2.60 |
|  | 6220538 | DOK2 | docking protein 2, 56kDa | 2.60 |
|  | 360187 | RIOK3 | RIO kinase 3 (yeast) | 2.58 |
|  | 1470215 | MAP3K8 | mitogen-activated protein kinase kinase kinase 8 | 2.57 |
|  | 1170072 | ZSWIM4 | zinc finger, SWIM-type containing 4 | 2.55 |
|  | 5870136 | CLIC3 | chloride intracellular channel 3 | 2.54 |
|  | 4880392 | KIAA1539 | KIAA1539 | 2.53 |
|  | 130609 | FCGBP | Fc fragment of IgG binding protein | 2.52 |
|  | 6330224 | ADCY4 | adenylate cyclase 4 | 2.49 |
|  | 1660296 | ID2 | inhibitor of DNA binding 2, dominant negative helix-loop-helix protein | 2.48 |
|  | 3400019 | RGS2 | regulator of G-protein signaling 2, 24kDa | 2.47 |
|  | 840554 | RYBP | RING1 and YY1 binding protein | 2.46 |
|  | 3400450 | CHKB | choline kinase beta | 2.45 |
|  | 1740136 | SLC38A2 | solute carrier family 38, member 2 | 2.41 |
|  | 2510523 | RBCK1 | RanBP-type and C3HC4-type zinc finger containing 1 | 2.41 |
|  | 650020 | SYNJ1 | synaptojanin 1 | 2.39 |
|  | 6620201 | KLHL24 | kelch-like 24 (Drosophila) | 2.39 |
|  | 4390450 | SGK1 | serum/glucocorticoid regulated kinase 1 | 2.37 |
|  | 460164 | LOC642567 | similar to ferritin, heavy polypeptide 1 | 2.35 |
|  | 3830653 | DNAJB2 | DnaJ (Hsp40) homolog, subfamily B, member 2 | 2.34 |
|  | 5310411 | H2AFJ | H2A histone family, member J | 2.34 |
|  | 3170431 | LRCH4 | leucine-rich repeats and calponin homology (CH) domain containing 4 | 2.34 |
|  | 6450139 | FTHL3P | ferritin, heavy polypeptide-like 3 pseudogene | 2.32 |
|  | 4220193 | SIGIRR | single immunoglobulin and toll-interleukin 1 receptor (TIR) domain | 2.29 |
|  | 5310053 | LTB | lymphotoxin beta (TNF superfamily, member 3) | 2.29 |
|  | 520463 | GPER | G protein-coupled estrogen receptor 1 | 2.28 |
|  | 3990259 | TMEM91 | transmembrane protein 91 | 2.27 |
|  | 4230437 | SLC35C2 | solute carrier family 35, member C2 | 2.27 |
|  | 5670577 | TSPAN31 | tetraspanin 31 | 2.26 |
|  | 1240300 | ENO3 | enolase 3 (beta, muscle) | 2.26 |
|  | 6350632 | TSC22D3 | TSC22 domain family, member 3 | 2.26 |
|  | 4250427 | ZNF493 | zinc finger protein 493 | 2.25 |
|  | 3360646 | MVP | major vault protein | 2.23 |
|  | 380437 | MGC52282 | transmembrane protease, serine pseudogene | 2.23 |
|  | 1510020 | LRCH4 | leucine-rich repeats and calponin homology (CH) domain containing 4 | 2.23 |
|  | 2470348 | NFKBIZ | nuclear factor of kappa light polypeptide gene enhancer in B-cells inhibitor, zeta | 2.22 |
|  | 780504 | TRGV3 | T cell receptor gamma variable 3 | 2.21 |
|  | 4010008 | SNORD43 | small nucleolar RNA, C/D box 43 | 2.21 |
|  | 5870594 | RAB33B | RAB33B, member RAS oncogene family | 2.20 |
|  | 3780270 | HBP1 | HMG-box transcription factor 1 | 2.20 |
|  | 2650564 | RARRES3 | retinoic acid receptor responder (tazarotene induced) 3 | 2.20 |
|  | 6330132 | ISG20 | interferon stimulated exonuclease gene 20kDa | 2.20 |
|  | 6620609 | ABTB1 | ankyrin repeat and BTB (POZ) domain containing 1 | 2.18 |
|  | 2600747 | IFIT2 | interferon-induced protein with tetratricopeptide repeats 2 | 2.18 |
|  | 270593 | GPER | G protein-coupled estrogen receptor 1 | 2.17 |
|  | 1440615 | OTOF | otoferlin | 2.17 |
|  | 6200692 | FOXO3 | forkhead box O3 | 2.17 |
|  | 6420386 | GPSM3 | G-protein signaling modulator 3 (AGS3-like, C, elegans) | 2.17 |
|  | 6270615 | GSDMB | gasdermin B | 2.15 |
|  | 5890136 | KIAA0913 | KIAA0913 | 2.15 |
|  | 5670719 | IL10RB | interleukin 10 receptor, beta | 2.15 |
|  | 5220035 | TUFT1 | tuftelin 1 | 2.14 |
|  | 1780719 | PTGES3 | prostaglandin E synthase 3 (cytosolic) | -2.14 |
|  | 6760504 | SEH1L | SEH1-like (S, cerevisiae) | -2.14 |
|  | 7330301 | DHFR | dihydrofolate reductase | -2.14 |
|  | 5390100 | IARS | isoleucyl-tRNA synthetase | -2.15 |
|  | 2190411 | PRKCB | protein kinase C, beta | -2.15 |
|  | 5490097 | UBAP2 | ubiquitin associated protein 2 | -2.15 |
|  | 5860605 | PDCD6IP | programmed cell death 6 interacting protein | -2.15 |
|  | 1260309 | IGLL1 | immunoglobulin lambda-like polypeptide 1 | -2.15 |
|  | 4830427 | PPIL5 | peptidylprolyl isomerase (cyclophilin)-like 5 | -2.16 |
|  | 6840577 | KPNB1 | karyopherin (importin) beta 1 | -2.16 |
|  | 3610259 | TUBB | tubulin, beta | -2.16 |
|  | 3460669 | PRMT1 | protein arginine methyltransferase 1 | -2.16 |
|  | 6200390 | PRIM1 | primase, DNA, polypeptide 1 (49kDa) | -2.17 |
|  | 7380181 | ELOVL6 | ELOVL family member 6, elongation of long chain fatty acids (FEN1/Elo2, SUR4/Elo3-like, yeast) | -2.17 |
|  | 3800520 | PSPH | phosphoserine phosphatase | -2.17 |
|  | 670647 | BMP2K | BMP2 inducible kinase | -2.17 |
|  | 5270367 | CTSC | cathepsin C | -2.17 |
|  | 4560270 | PTPLB | protein tyrosine phosphatase-like (proline instead of catalytic arginine), member b | -2.17 |
|  | 2450156 | XBP1 | X-box binding protein 1 | -2.17 |
|  | 990725 | DONSON | downstream neighbor of SON | -2.17 |
|  | 2810730 | PPHLN1 | periphilin 1 | -2.17 |
|  | 6370273 | CTPS | CTP synthase | -2.18 |
|  | 110195 | HSP90AB1 | heat shock protein 90kDa alpha (cytosolic), class B member 1 | -2.18 |
|  | 2470020 | TNPO1 | transportin 1 | -2.18 |
|  | 1850035 | PPIA | peptidylprolyl isomerase A (cyclophilin A) | -2.18 |
|  | 4040376 | LARP1 | La ribonucleoprotein domain family, member 1 | -2.19 |
|  | 5270315 | WDR12 | WD repeat domain 12 | -2.19 |
|  | 1090543 | ABCE1 | ATP-binding cassette, sub-family E (OABP), member 1 | -2.19 |
|  | 5310152 | ABCE1 | ATP-binding cassette, sub-family E (OABP), member 1 | -2.19 |
|  | 1570162 | TBL2 | transducin (beta)-like 2 | -2.19 |
|  | 1850593 | AP1G1 | adaptor-related protein complex 1, gamma 1 subunit | -2.19 |
|  | 2140762 | YWHAE | tyrosine 3-monooxygenase/tryptophan 5-monooxygenase activation protein, epsilon polypeptide | -2.19 |
|  | 5720128 | COQ2 | coenzyme Q2 homolog, prenyltransferase (yeast) | -2.20 |
|  | 3780019 | HIGD1A | HIG1 domain family, member 1A | -2.20 |
|  | 5050053 | TXNDC5 | thioredoxin domain containing 5 (endoplasmic reticulum) | -2.20 |
|  | 5700739 | LARP7 | La ribonucleoprotein domain family, member 7 | -2.20 |
|  | 7050204 | KIAA1737 | KIAA1737 | -2.20 |
|  | 650278 | SNORA70 | small nucleolar RNA, H/ACA box 70 | -2.20 |
|  | 1500152 | ACP1 | acid phosphatase 1, soluble | -2.20 |
|  | 650722 | ASCC3 | activating signal cointegrator 1 complex subunit 3 | -2.21 |
|  | 2600066 | SUMO2 | SMT3 suppressor of mif two 3 homolog 2 (S, cerevisiae) | -2.21 |
|  | 450598 | PHF5A | PHD finger protein 5A | -2.21 |
|  | 3120767 | CNIH | cornichon homolog (Drosophila) | -2.21 |
|  | 6480064 | POLR1E | polymerase (RNA) I polypeptide E, 53kDa | -2.22 |
|  | 2510039 | ENDOD1 | endonuclease domain containing 1 | -2.22 |
|  | 3120671 | MST075 | MSTP075 | -2.23 |
|  | 4540039 | C1orf109 | chromosome 1 open reading frame 109 | -2.23 |
|  | 10440 | MARS2 | methionyl-tRNA synthetase 2, mitochondrial | -2.23 |
|  | 7160678 | ESD | esterase D/formylglutathione hydrolase | -2.23 |
|  | 2360471 | H2AFY | H2A histone family, member Y | -2.24 |
|  | 3130632 | POLR2B | polymerase (RNA) II (DNA directed) polypeptide B, 140kDa | -2.24 |
|  | 4120437 | RRS1 | RRS1 ribosome biogenesis regulator homolog (S, cerevisiae) | -2.25 |
|  | 2480612 | DCTD | dCMP deaminase | -2.25 |
|  | 4570026 | CASP2 | caspase 2, apoptosis-related cysteine peptidase | -2.25 |
|  | 4830500 | IPO4 | importin 4 | -2.25 |
|  | 4540435 | HN1 | hematological and neurological expressed 1 | -2.25 |
|  | 6420349 | PTPLAD1 | protein tyrosine phosphatase-like A domain containing 1 | -2.25 |
|  | 5220392 | NA | NA | -2.25 |
|  | 4570598 | KEAP1 | kelch-like ECH-associated protein 1 | -2.25 |
|  | 1740139 | USP10 | ubiquitin specific peptidase 10 | -2.25 |
|  | 1770646 | EXO1 | exonuclease 1 | -2.26 |
|  | 160204 | RPP40 | ribonuclease P/MRP 40kDa subunit | -2.26 |
|  | 460711 | BAT2D1 | BAT2 domain containing 1 | -2.26 |
|  | 4780561 | NA | NA | -2.27 |
|  | 10546 | RPS24 | ribosomal protein S24 | -2.27 |
|  | 160397 | CSPG5 | chondroitin sulfate proteoglycan 5 (neuroglycan C) | -2.27 |
|  | 1660270 | MTHFD1 | methylenetetrahydrofolate dehydrogenase (NADP+ dependent) 1, methenyltetrahydrofolate cyclohydrolase, formyltetrahydrofolate synthetase | -2.27 |
|  | 5050608 | TIMM23 | translocase of inner mitochondrial membrane 23 homolog (yeast) | -2.27 |
|  | 150543 | FAM83D | family with sequence similarity 83, member D | -2.28 |
|  | 2100484 | STAT3 | signal transducer and activator of transcription 3 (acute-phase response factor) | -2.28 |
|  | 990184 | PSMD6 | proteasome (prosome, macropain) 26S subunit, non-ATPase, 6 | -2.28 |
|  | 2710341 | PRNP | prion protein | -2.28 |
|  | 1050706 | CDC2 | cell division cycle 2, G1 to S and G2 to M | -2.29 |
|  | 3780056 | GGCT | gamma-glutamyl cyclotransferase | -2.29 |
|  | 4780450 | ATP5C1 | ATP synthase, H+ transporting, mitochondrial F1 complex, gamma polypeptide 1 | -2.29 |
|  | 3310056 | NOMO1 | NODAL modulator 1 | -2.29 |
|  | 3870594 | IFI16 | interferon, gamma-inducible protein 16 | -2.29 |
|  | 7610286 | HPRT1 | hypoxanthine phosphoribosyltransferase 1 | -2.30 |
|  | 6200202 | APIP | APAF1 interacting protein | -2.30 |
|  | 6650228 | LOC100133788 | similar to SMT3B protein | -2.30 |
|  | 4390113 | UNC84A | unc-84 homolog A (C, elegans) | -2.30 |
|  | 2630687 | CHPT1 | choline phosphotransferase 1 | -2.31 |
|  | 5960709 | APITD1 | apoptosis-inducing, TAF9-like domain 1 | -2.31 |
|  | 5720445 | SLC35F2 | solute carrier family 35, member F2 | -2.31 |
|  | 7570050 | HMGB3 | high-mobility group box 3 | -2.31 |
|  | 2750446 | RPL7A | ribosomal protein L7a | -2.32 |
|  | 5310634 | FASN | fatty acid synthase | -2.33 |
|  | 2940164 | PKMYT1 | protein kinase, membrane associated tyrosine/threonine 1 | -2.33 |
|  | 1430706 | PPHLN1 | periphilin 1 | -2.33 |
|  | 4900088 | CTBP1 | C-terminal binding protein 1 | -2.33 |
|  | 5080603 | ACTL6A | actin-like 6A | -2.34 |
|  | 450348 | GNG10 | guanine nucleotide binding protein (G protein), gamma 10 | -2.34 |
|  | 780228 | RBM3 | RNA binding motif (RNP1, RRM) protein 3 | -2.35 |
|  | 3890747 | RPL4P5 | ribosomal protein L4 pseudogene 5 | -2.36 |
|  | 6280040 | YWHAG | tyrosine 3-monooxygenase/tryptophan 5-monooxygenase activation protein, gamma polypeptide | -2.36 |
|  | 780240 | C12orf24 | chromosome 12 open reading frame 24 | -2.37 |
|  | 6660131 | TSPAN13 | tetraspanin 13 | -2.37 |
|  | 5420482 | AIF1L | allograft inflammatory factor 1-like | -2.37 |
|  | 5570196 | ACTR2 | ARP2 actin-related protein 2 homolog (yeast) | -2.37 |
|  | 2970195 | DDX3X | DEAD (Asp-Glu-Ala-Asp) box polypeptide 3, X-linked | -2.38 |
|  | 6290358 | TPX2 | TPX2, microtubule-associated, homolog (Xenopus laevis) | -2.39 |
|  | 6110561 | MRPS27 | mitochondrial ribosomal protein S27 | -2.39 |
|  | 6580685 | MCM10 | minichromosome maintenance complex component 10 | -2.39 |
|  | 3360377 | UTP14A | UTP14, U3 small nucleolar ribonucleoprotein, homolog A (yeast) | -2.39 |
|  | 7200270 | CHEK1 | CHK1 checkpoint homolog (S, pombe) | -2.40 |
|  | 520224 | C3orf26 | chromosome 3 open reading frame 26 | -2.40 |
|  | 50348 | ALS2CR4 | amyotrophic lateral sclerosis 2 (juvenile) chromosome region, candidate 4 | -2.40 |
|  | 520577 | TIMM23B | translocase of inner mitochondrial membrane 23 homolog B (yeast) | -2.40 |
|  | 5080746 | HEATR3 | HEAT repeat containing 3 | -2.41 |
|  | 6480609 | RRP1B | ribosomal RNA processing 1 homolog B (S, cerevisiae) | -2.41 |
|  | 3840131 | NUP153 | nucleoporin 153kDa | -2.41 |
|  | 2070730 | NGDN | neuroguidin, EIF4E binding protein | -2.42 |
|  | 4730747 | IGLL1 | immunoglobulin lambda-like polypeptide 1 | -2.42 |
|  | 3710537 | AHCY | S-adenosylhomocysteine hydrolase | -2.42 |
|  | 4120278 | KIF23 | kinesin family member 23 | -2.42 |
|  | 7330753 | ACAT2 | acetyl-Coenzyme A acetyltransferase 2 | -2.43 |
|  | 450609 | IGLL3 | immunoglobulin lambda-like polypeptide 3 | -2.43 |
|  | 3120458 | NCL | nucleolin | -2.43 |
|  | 2510279 | NUP62 | nucleoporin 62kDa | -2.44 |
|  | 5390494 | EIF4B | eukaryotic translation initiation factor 4B | -2.44 |
|  | 870546 | MAD2L1 | MAD2 mitotic arrest deficient-like 1 (yeast) | -2.45 |
|  | 3310301 | YBX1 | Y box binding protein 1 | -2.45 |
|  | 2600431 | SLC43A3 | solute carrier family 43, member 3 | -2.45 |
|  | 5260014 | CDKN3 | cyclin-dependent kinase inhibitor 3 | -2.47 |
|  | 2680446 | ARF3 | ADP-ribosylation factor 3 | -2.47 |
|  | 580196 | SEH1L | SEH1-like (S, cerevisiae) | -2.47 |
|  | 4540370 | RAB7L1 | RAB7, member RAS oncogene family-like 1 | -2.47 |
|  | 4850253 | TNFRSF8 | tumor necrosis factor receptor superfamily, member 8 | -2.48 |
|  | 5570292 | PRDX3 | peroxiredoxin 3 | -2.48 |
|  | 2940066 | LOC645691 | similar to heterogeneous nuclear ribonucleoprotein A1 | -2.48 |
|  | 5260047 | FABP5L7 | fatty acid binding protein 5-like 7 | -2.49 |
|  | 830593 | VIM | vimentin | -2.49 |
|  | 5670112 | ANKRD27 | ankyrin repeat domain 27 (VPS9 domain) | -2.50 |
|  | 2970332 | WEE1 | WEE1 homolog (S, pombe) | -2.52 |
|  | 5490433 | ADAR | adenosine deaminase, RNA-specific | -2.52 |
|  | 4610047 | RANBP1 | RAN binding protein 1 | -2.54 |
|  | 3120307 | ZWINT | ZW10 interactor | -2.54 |
|  | 2900039 | XRCC5 | X-ray repair complementing defective repair in Chinese hamster cells 5 (double-strand-break rejoining) | -2.54 |
|  | 5720360 | STARD7 | StAR-related lipid transfer (START) domain containing 7 | -2.55 |
|  | 4010097 | FBXO5 | F-box protein 5 | -2.55 |
|  | 6510435 | MORF4L2 | mortality factor 4 like 2 | -2.55 |
|  | 6940025 | MRPL39 | mitochondrial ribosomal protein L39 | -2.56 |
|  | 5690095 | GDF10 | growth differentiation factor 10 | -2.56 |
|  | 3850121 | EEF1A1 | eukaryotic translation elongation factor 1 alpha 1 | -2.56 |
|  | 2650608 | CCNA2 | cyclin A2 | -2.57 |
|  | 610243 | THOC3 | THO complex 3 | -2.57 |
|  | 5820020 | PRDX3 | peroxiredoxin 3 | -2.57 |
|  | 460750 | PDCL3 | phosducin-like 3 | -2.58 |
|  | 4780521 | PRPS1 | phosphoribosyl pyrophosphate synthetase 1 | -2.58 |
|  | 2100273 | EEF1A1 | eukaryotic translation elongation factor 1 alpha 1 | -2.58 |
|  | 7400286 | HMGA1 | high mobility group AT-hook 1 | -2.59 |
|  | 5820202 | LOC644131 | similar to chaperonin containing TCP1, subunit 8 (theta) | -2.59 |
|  | 5360646 | LOC652826 | similar to 26S protease regulatory subunit 6B (MIP224) (MB67-interacting protein) (TAT-binding protein 7) (TBP-7) | -2.59 |
|  | 6290437 | MTHFD1L | methylenetetrahydrofolate dehydrogenase (NADP+ dependent) 1-like | -2.60 |
|  | 3370487 | HNRNPAB | heterogeneous nuclear ribonucleoprotein A/B | -2.60 |
|  | 2630711 | MCM4 | minichromosome maintenance complex component 4 | -2.60 |
|  | 2480291 | SNRPN | small nuclear ribonucleoprotein polypeptide N | -2.60 |
|  | 1820398 | HNRNPAB | heterogeneous nuclear ribonucleoprotein A/B | -2.61 |
|  | 7320170 | LOC399491 | LOC399491 protein | -2.61 |
|  | 5390037 | hCG_1781062 | hCG1781062 | -2.61 |
|  | 5090754 | KIAA0101 | KIAA0101 | -2.62 |
|  | 1980424 | LOC647349 | similar to Adapter-related protein complex 3 sigma 1 subunit (Sigma-adaptin 3a) (AP-3 complex sigma-3A subunit) (Sigma-3A-adaptin) | -2.63 |
|  | 1010168 | PSMG1 | proteasome (prosome, macropain) assembly chaperone 1 | -2.64 |
|  | 1940632 | NCAPG2 | non-SMC condensin II complex, subunit G2 | -2.65 |
|  | 2970292 | SMC3 | structural maintenance of chromosomes 3 | -2.67 |
|  | 730092 | SFXN4 | sideroflexin 4 | -2.67 |
|  | 4200220 | RNF4 | ring finger protein 4 | -2.68 |
|  | 5910364 | TYMS | thymidylate synthetase | -2.69 |
|  | 3800474 | ZWINT | ZW10 interactor | -2.69 |
|  | 7320424 | HNRPA1L-2 | heterogeneous nuclear ribonucleoprotein A1 pseudogene | -2.69 |
|  | 2640554 | EEF1B2 | eukaryotic translation elongation factor 1 beta 2 | -2.69 |
|  | 360424 | SDHA | succinate dehydrogenase complex, subunit A, flavoprotein (Fp) | -2.69 |
|  | 430025 | CSNK2A1 | casein kinase 2, alpha 1 polypeptide | -2.69 |
|  | 5720746 | CDV3 | CDV3 homolog (mouse) | -2.70 |
|  | 7040327 | ALDH5A1 | aldehyde dehydrogenase 5 family, member A1 | -2.73 |
|  | 2190474 | LRCH3 | leucine-rich repeats and calponin homology (CH) domain containing 3 | -2.73 |
|  | 4150048 | FABP5L7 | fatty acid binding protein 5-like 7 | -2.75 |
|  | 7550133 | TSR1 | TSR1, 20S rRNA accumulation, homolog (S, cerevisiae) | -2.76 |
|  | 3610343 | NBN | nibrin | -2.76 |
|  | 7210435 | RFC4 | replication factor C (activator 1) 4, 37kDa | -2.77 |
|  | 4230327 | STK35 | serine/threonine kinase 35 | -2.80 |
|  | 4290279 | ACLY | ATP citrate lyase | -2.80 |
|  | 3990273 | LOC728188 | similar to phosphoglycerate mutase processed protein | -2.81 |
|  | 3170446 | NUDT21 | nudix (nucleoside diphosphate linked moiety X)-type motif 21 | -2.83 |
|  | 6770563 | LOC441228 | similar to Exportin-T (tRNA exportin) (Exportin(tRNA)) | -2.84 |
|  | 540725 | HNRNPH1 | heterogeneous nuclear ribonucleoprotein H1 (H) | -2.84 |
|  | 2510678 | PKMYT1 | protein kinase, membrane associated tyrosine/threonine 1 | -2.85 |
|  | 6060653 | DNMT3B | DNA (cytosine-5-)-methyltransferase 3 beta | -2.87 |
|  | 5220358 | ACTL6A | actin-like 6A | -2.87 |
|  | 4830343 | ORC6L | origin recognition complex, subunit 6 like (yeast) | -2.87 |
|  | 5870307 | CSDAP1 | cold shock domain protein A pseudogene 1 | -2.88 |
|  | 1980564 | EIF3M | eukaryotic translation initiation factor 3, subunit M | -2.93 |
|  | 1010598 | ATP5F1 | ATP synthase, H+ transporting, mitochondrial F0 complex, subunit B1 | -2.95 |
|  | 2690047 | ARL6IP1 | ADP-ribosylation factor-like 6 interacting protein 1 | -2.95 |
|  | 3290435 | TFDP1 | transcription factor Dp-1 | -2.96 |
|  | 4390195 | HMGN1 | high-mobility group nucleosome binding domain 1 | -2.96 |
|  | 3130356 | CDV3 | CDV3 homolog (mouse) | -2.98 |
|  | 6250053 | PTTG1IP | pituitary tumor-transforming 1 interacting protein | -2.98 |
|  | 4590241 | CNOT7 | CCR4-NOT transcription complex, subunit 7 | -2.99 |
|  | 7650524 | BCAT1 | branched chain aminotransferase 1, cytosolic | -3.00 |
|  | 3450682 | MYCN | v-myc myelocytomatosis viral related oncogene, neuroblastoma derived (avian) | -3.00 |
|  | 5890500 | LOC728188 | similar to phosphoglycerate mutase processed protein | -3.00 |
|  | 5570152 | ATP1A1 | ATPase, Na+/K+ transporting, alpha 1 polypeptide | -3.04 |
|  | 6290494 | MCM3 | minichromosome maintenance complex component 3 | -3.06 |
|  | 3460121 | TOMM20 | translocase of outer mitochondrial membrane 20 homolog (yeast) | -3.06 |
|  | 5270167 | GNL3 | guanine nucleotide binding protein-like 3 (nucleolar) | -3.06 |
|  | 4280133 | UFM1 | ubiquitin-fold modifier 1 | -3.07 |
|  | 6020402 | LOC646347 | similar to spermine synthase | -3.10 |
|  | 4900465 | RUVBL1 | RuvB-like 1 (E, coli) | -3.10 |
|  | 1070612 | DVL3 | dishevelled, dsh homolog 3 (Drosophila) | -3.10 |
|  | 3420593 | LMNB1 | lamin B1 | -3.12 |
|  | 6350053 | NUSAP1 | nucleolar and spindle associated protein 1 | -3.13 |
|  | 4490341 | SMS | spermine synthase | -3.14 |
|  | 6620689 | MTHFD2 | methylenetetrahydrofolate dehydrogenase (NADP+ dependent) 2, methenyltetrahydrofolate cyclohydrolase | -3.15 |
|  | 5820619 | HNRNPK | heterogeneous nuclear ribonucleoprotein K | -3.15 |
|  | 6380082 | NAT5 | N-acetyltransferase 5 (GCN5-related, putative) | -3.16 |
|  | 1010719 | FEN1 | flap structure-specific endonuclease 1 | -3.17 |
|  | 3290291 | SLBP | stem-loop binding protein | -3.23 |
|  | 5670468 | EIF5 | eukaryotic translation initiation factor 5 | -3.31 |
|  | 6220148 | LYAR | Ly1 antibody reactive homolog (mouse) | -3.32 |
|  | 3890008 | CYCS | cytochrome c, somatic | -3.33 |
|  | 4540397 | EIF4H | eukaryotic translation initiation factor 4H | -3.38 |
|  | 6110168 | LOC728188 | similar to phosphoglycerate mutase processed protein | -3.39 |
|  | 4060446 | LOC644604 | similar to rCG25445 | -3.41 |
|  | 7200524 | IGLL1 | immunoglobulin lambda-like polypeptide 1 | -3.53 |
|  | 2360608 | HNRNPL | heterogeneous nuclear ribonucleoprotein L | -3.71 |
|  | 2630561 | RPL6 | ribosomal protein L6 | -3.80 |
|  | 5900600 | CNBP | CCHC-type zinc finger, nucleic acid binding protein | -3.84 |
|  | 2450136 | PSMG1 | proteasome (prosome, macropain) assembly chaperone 1 | -3.85 |
|  | 2470187 | CS | citrate synthase | -3.85 |
|  | 770437 | TIMM23B | translocase of inner mitochondrial membrane 23 homolog B (yeast) | -3.89 |
|  | 7000735 | RANBP1 | RAN binding protein 1 | -4.00 |
|  | 2850020 | DHRS2 | dehydrogenase/reductase (SDR family) member 2 | -4.17 |
|  | 3420400 | KPNA2 | karyopherin alpha 2 (RAG cohort 1, importin alpha 1) | -4.18 |
|  | 4230196 | KPNA2 | karyopherin alpha 2 (RAG cohort 1, importin alpha 1) | -4.48 |
|  | 460286 | THOC4 | THO complex 4 | -4.50 |
|  | 4390315 | HNRNPA1P2 | heterogeneous nuclear ribonucleoprotein A1 pseudogene 2 | -4.77 |
|  | 2750719 | DDX21 | DEAD (Asp-Glu-Ala-Asp) box polypeptide 21 | -4.91 |
|  | 4890671 | DHRS2 | dehydrogenase/reductase (SDR family) member 2 | -5.68 |
|  | 4880646 | ACTBL3 | actin, beta-like 3 | -5.70 |
|  | 3940592 | PGAM1 | phosphoglycerate mutase 1 (brain) | -5.76 |
|  | 5270730 | ACTB | actin, beta | -6.50 |

**Table S3.** Complete list of Signaling pathways with corresponding genes affected by treatment of CCRF-CEM cells with guieranone A.

| **Ingenuity Canonical Pathways** | **-log(p-value)** | **Ratio** | **Molecules** |
| --- | --- | --- | --- |
| Cell Cycle: G2/M DNA Damage Checkpoint Regulation | 4,44E00 | 1,25E-01 | YWHAG, YWHAE, WEE1, PKMYT1, CDK1, CHEK1 |
| One Carbon Pool by Folate | 4,09E00 | 1,32E-01 | TYMS, MTHFD2, DHFR, MTHFD1, MTHFD1L |
| ATM Signaling | 3,96E00 | 1,11E-01 | SMC3, JUN, GADD45B, CDK1, CHEK1, NBN |
| RAN Signaling | 3,93E00 | 1,74E-01 | KPNB1, KPNA2, TNPO1, RANBP1 |
| Aryl Hydrocarbon Receptor Signaling | 3,04E00 | 5,3E-02 | CCNA2, JUN, PTGES3 (includes EG:10728), TFDP1, HSP90AB1, DHFR, ALDH5A1, CHEK1 |
| Role of CHK Proteins in Cell Cycle Checkpoint Control | 2,88E00 | 1,14E-01 | RFC4, CDK1, CHEK1, NBN |
| Mitotic Roles of Polo-Like Kinase | 2,83E00 | 8,47E-02 | KIF23, HSP90AB1, WEE1, PKMYT1, CDK1 |
| Hereditary Breast Cancer Signaling | 2,75E00 | 5,51E-02 | GADD45B, RFC4, WEE1, POLR2B, CDK1, CHEK1, NBN |
| 14-3-3-mediated Signaling | 2,72E00 | 5,79E-02 | YWHAG, JUN, YWHAE, VIM, PDCD6IP, TUBB, PRKCB |
| Huntington's Disease Signaling | 2,69E00 | 4,13E-02 | SDHA, JUN, HSPA1A, SGK1, CASP2, HSPA6, CYCS (includes EG:54205), POLR2B, GNG10, PRKCB |
| Mismatch Repair in Eukaryotes | 2,66E00 | 1,25E-01 | RFC4, FEN1, EXO1 |
| Mechanisms of Viral Exit from Host Cells | 2,56E00 | 8,89E-02 | ACTB, PDCD6IP, LMNB1, PRKCB |
| ERK5 Signaling | 2,55E00 | 7,35E-02 | YWHAG, YWHAE, SGK1, FOXO3, MAP3K8 |
| PDGF Signaling | 2,26E00 | 6,33E-02 | JUN, ACP1, CSNK2A1, STAT3, PRKCB |
| Glyoxylate and Dicarboxylate Metabolism | 2,24E00 | 3,64E-02 | CS, MTHFD2, MTHFD1, MTHFD1L |
| Cell Cycle Control of Chromosomal Replication | 2,15E00 | 9,68E-02 | MCM3, ORC6L, MCM4 |
| Methionine Metabolism | 2,15E00 | 5,13E-02 | DNMT3B, SMS, MARS2, AHCY |
| Purine Metabolism | 2,03E00 | 2,78E-02 | ATP5C1, PRIM1, PRPS1, ADCY4, POLR1E, PDE1B, HPRT1, PSMD6, RUVBL1, POLR2B, ADAR, ATP5F1 |
| Aldosterone Signaling in Epithelial Cells | 1,95E00 | 4,09E-02 | HSP90AB1, HSPA1A, SGK1, HSPA6, DNAJB2 (includes EG:3300), PRKCB, AHCY |
| IGF-1 Signaling | 1,94E00 | 5,1E-02 | YWHAG, JUN, YWHAE, FOXO3, CSNK2A1 |
| Activation of IRF by Cytosolic Pattern Recognition Receptors | 1,91E00 | 5,71E-02 | JUN, STAT2, ADAR, IFIT2 |
| NRF2-mediated Oxidative Stress Response | 1,79E00 | 3,68E-02 | HMOX1, JUN, KEAP1, ACTB, DNAJB2 (includes EG:3300), PTPLAD1, PRKCB |
| DNA Double-Strand Break Repair by Non-Homologous End Joining | 1,76E00 | 1,05E-01 | XRCC5, NBN |
| IL-10 Signaling | 1,67E00 | 5,13E-02 | HMOX1, JUN, IL10RB, STAT3 |
| Granzyme B Signaling | 1,65E00 | 1,25E-01 | CYCS (includes EG:54205), LMNB1 |
| Cyclins and Cell Cycle Regulation | 1,57E00 | 4,6E-02 | CCNA2, TFDP1, WEE1, CDK1 |
| TNFR1 Signaling | 1,5E00 | 5,77E-02 | JUN, CASP2, CYCS (includes EG:54205) |
| Butanoate Metabolism | 1,47E00 | 3,12E-02 | SDHA, ACAT2, ALDH5A1, ELOVL6 |
| PI3K/AKT Signaling | 1,45E00 | 3,6E-02 | YWHAG, YWHAE, HSP90AB1, FOXO3, MAP3K8 |
| EGF Signaling | 1,43E00 | 5,77E-02 | JUN, CSNK2A1, STAT3 |
| Citrate Cycle | 1,43E00 | 5,26E-02 | SDHA, CS, ACLY |
| Glucocorticoid Receptor Signaling | 1,42E00 | 2,84E-02 | TSC22D3, JUN, PTGES3 (includes EG:10728), HSP90AB1, HSPA1A, HSPA6, STAT3, POLR2B |
| CD27 Signaling in Lymphocytes | 1,41E00 | 5,36E-02 | JUN, CYCS (includes EG:54205), MAP3K8 |
| Xenobiotic Metabolism Signaling | 1,4E00 | 2,94E-02 | HMOX1, PTGES3 (includes EG:10728), HSP90AB1, KEAP1, MAP3K8, ALDH5A1, ESD, PRKCB |
| RAR Activation | 1,39E00 | 3,31E-02 | PRMT1, JUN, RPL7A (includes EG:6130), ADCY4, CSNK2A1, PRKCB |
| Valine, Leucine and Isoleucine Biosynthesis | 1,32E00 | 4,65E-02 | BCAT1, IARS |
| Role of BRCA1 in DNA Damage Response | 1,31E00 | 4,92E-02 | RFC4, CHEK1, NBN |
| VEGF Signaling | 1,3E00 | 4E-02 | YWHAE, ACTB, FOXO3, PRKCB |
| Valine, Leucine and Isoleucine Degradation | 1,29E00 | 3,81E-02 | BCAT1, ACAT2, ALDH5A1, ELOVL6 |
| p53 Signaling | 1,27E00 | 4,04E-02 | JUN, GADD45B, GNL3, CHEK1 |
| Fcγ Receptor-mediated Phagocytosis in Macrophages and Monocytes | 1,26E00 | 3,96E-02 | HMOX1, ACTR2, ACTB, PRKCB |
| Thrombopoietin Signaling | 1,25E00 | 4,76E-02 | JUN, STAT3, PRKCB |
| IL-22 Signaling | 1,23E00 | 7,14E-02 | IL10RB, STAT3 |
| Myc Mediated Apoptosis Signaling | 1,22E00 | 4,76E-02 | YWHAG, YWHAE, CYCS (includes EG:54205) |
| HGF Signaling | 1,17E00 | 3,74E-02 | JUN, MAP3K8, STAT3, PRKCB |
| Pyrimidine Metabolism | 1,14E00 | 2,64E-02 | TYMS, PRIM1, POLR1E, POLR2B, DCTD, CTPS |
| Clathrin-mediated Endocytosis Signaling | 1,06E00 | 2,96E-02 | ACTR2, SYNJ1, ACTB, CSNK2A1, ITGB7 |
| PPARα/RXRα Activation | 1,04E00 | 2,89E-02 | JUN, HSP90AB1, FASN, ADCY4, PRKCB |
| Renin-Angiotensin Signaling | 1,04E00 | 3,23E-02 | JUN, ADCY4, STAT3, PRKCB |
| Androgen Signaling | 1,04E00 | 2,84E-02 | JUN, POLR2B, GNG10, PRKCB |
| IL-3 Signaling | 1,03E00 | 3,95E-02 | JUN, STAT3, PRKCB |
| Cell Cycle Regulation by BTG Family Proteins | 1,03E00 | 5,56E-02 | PRMT1, CNOT7 |
| CCR5 Signaling in Macrophages | 1,02E00 | 3,33E-02 | JUN, GNG10, PRKCB |
| Glycolysis/Gluconeogenesis | 1,02E00 | 2,92E-02 | DHRS2 (includes EG:10202), ENO3, PGAM1, ALDH5A1 |
| Histidine Metabolism | 1,01E00 | 2,52E-02 | PRPS1, ALDH5A1, ELOVL6 |
| Prolactin Signaling | 9,94E-01 | 3,75E-02 | JUN, STAT3, PRKCB |
| Lysine Degradation | 9,69E-01 | 2,22E-02 | ACAT2, ALDH5A1, ELOVL6 |
| P2Y Purigenic Receptor Signaling Pathway | 9,21E-01 | 2,92E-02 | JUN, ADCY4, GNG10, PRKCB |
| GNRH Signaling | 9,12E-01 | 2,84E-02 | JUN, ADCY4, MAP3K8, PRKCB |
| Apoptosis Signaling | 9,09E-01 | 3,41E-02 | CASP2, CYCS (includes EG:54205), CDK1 |
| Endothelin-1 Signaling | 8,48E-01 | 2,62E-02 | HMOX1, JUN, ADCY4, CASP2, PRKCB |
| IL-6 Signaling | 7,94E-01 | 3E-02 | JUN, CSNK2A1, STAT3 |
| Cleavage and Polyadenylation of Pre-mRNA | 7,88E-01 | 8,33E-02 | NUDT21 |
| Virus Entry via Endocytic Pathways | 7,85E-01 | 3,03E-02 | ACTB, ITGB7, PRKCB |
| IL-1 Signaling | 7,85E-01 | 2,8E-02 | JUN, ADCY4, GNG10 |
| Relaxin Signaling | 7,81E-01 | 2,52E-02 | JUN, ADCY4, PDE1B, GNG10 |
| Breast Cancer Regulation by Stathmin1 | 7,63E-01 | 2,38E-02 | ADCY4, TUBB, CDK1, GNG10, PRKCB |
| Mitochondrial Dysfunction | 7,6E-01 | 2,41E-02 | SDHA, ATP5C1, PRDX3, CYCS (includes EG:54205) |
| α-Adrenergic Signaling | 7,57E-01 | 2,83E-02 | ADCY4, GNG10, PRKCB |
| DNA Double-Strand Break Repair by Homologous Recombination | 7,27E-01 | 5,88E-02 | NBN |
| Inositol Phosphate Metabolism | 7,18E-01 | 2,23E-02 | SYNJ1, SGK1, MAP3K8, CDK1 |
| Selenoamino Acid Metabolism | 7,13E-01 | 2,63E-02 | MARS2, AHCY |
| Protein Ubiquitination Pathway | 7,07E-01 | 2,19E-02 | HSP90AB1, HSPA1A, HSPA6, USP10, PSMD6, DNAJB2 (includes EG:3300) |
| RhoA Signaling | 6,81E-01 | 2,78E-02 | ACTR2, LPAR2, ACTB |
| Germ Cell-Sertoli Cell Junction Signaling | 6,78E-01 | 2,41E-02 | KEAP1, ACTB, MAP3K8, TUBB |
| IL-2 Signaling | 6,78E-01 | 3,28E-02 | JUN, CSNK2A1 |
| Death Receptor Signaling | 6,78E-01 | 3,17E-02 | CASP2, CYCS (includes EG:54205) |
| cAMP-mediated Signaling | 6,77E-01 | 2,3E-02 | RGS2, GPER, ADCY4, PDE1B, STAT3 |
| Glycerophospholipid Metabolism | 6,72E-01 | 2,11E-02 | HMOX1, CHPT1, CHKB, ELOVL6 |
| Lymphotoxin β Receptor Signaling | 6,67E-01 | 3,17E-02 | LTB, CYCS (includes EG:54205) |
| CXCR4 Signaling | 6,66E-01 | 2,35E-02 | JUN, ADCY4, GNG10, PRKCB |
| Pancreatic Adenocarcinoma Signaling | 6,65E-01 | 2,5E-02 | HMOX1, TFDP1, STAT3 |
| NF-κB Signaling | 6,54E-01 | 2,33E-02 | SIGIRR, CSNK2A1, MAP3K8, PRKCB |
| Endoplasmic Reticulum Stress Pathway | 6,52E-01 | 5,88E-02 | XBP1 |
| Synthesis and Degradation of Ketone Bodies | 6,52E-01 | 5,26E-02 | ACAT2 |
| CD40 Signaling | 6,36E-01 | 2,9E-02 | JUN, STAT3 |
| Parkinson's Signaling | 6,31E-01 | 5,56E-02 | CYCS (includes EG:54205) |
| IL-12 Signaling and Production in Macrophages | 6,28E-01 | 2,26E-02 | JUN, MAP3K8, PRKCB |
| Glycine, Serine and Threonine Metabolism | 6,28E-01 | 2,04E-02 | PSPH, CHKB, ELOVL6 |
| Molecular Mechanisms of Cancer | 6,21E-01 | 1,89E-02 | JUN, TFDP1, ADCY4, CYCS (includes EG:54205), CHEK1, PRKCB, NBN |
| fMLP Signaling in Neutrophils | 6,14E-01 | 2,33E-02 | ACTR2, GNG10, PRKCB |
| Nicotinate and Nicotinamide Metabolism | 6,14E-01 | 2,26E-02 | SGK1, MAP3K8, CDK1 |
| JAK/Stat Signaling | 6,06E-01 | 2,94E-02 | STAT2, STAT3 |
| GM-CSF Signaling | 5,96E-01 | 2,86E-02 | STAT3, PRKCB |
| Acute Phase Response Signaling | 5,96E-01 | 2,19E-02 | HMOX1, JUN, STAT3, HNRNPK |
| Ephrin Receptor Signaling | 5,96E-01 | 2,02E-02 | ACTR2, ACP1, STAT3, GNG10 |
| DNA Methylation and Transcriptional Repression Signaling | 5,91E-01 | 4,35E-02 | DNMT3B |
| Corticotropin Releasing Hormone Signaling | 5,86E-01 | 2,29E-02 | JUN, ADCY4, PRKCB |
| T Helper Cell Differentiation | 5,78E-01 | 2,86E-02 | IL10RB, STAT3 |
| Agrin Interactions at Neuromuscular Junction | 5,78E-01 | 2,94E-02 | JUN, ACTB |
| Hypoxia Signaling in the Cardiovascular System | 5,78E-01 | 2,9E-02 | JUN, HSP90AB1 |
| Macropinocytosis Signaling | 5,78E-01 | 2,63E-02 | ITGB7, PRKCB |
| Fatty Acid Biosynthesis | 5,73E-01 | 1,96E-02 | FASN |
| ILK Signaling | 5,69E-01 | 2,16E-02 | JUN, ACTB, VIM, ITGB7 |
| CREB Signaling in Neurons | 5,48E-01 | 1,99E-02 | ADCY4, POLR2B, GNG10, PRKCB |
| FLT3 Signaling in Hematopoietic Progenitor Cells | 5,44E-01 | 2,7E-02 | STAT2, STAT3 |
| Growth Hormone Signaling | 5,44E-01 | 2,67E-02 | STAT3, PRKCB |
| p70S6K Signaling | 5,42E-01 | 2,29E-02 | YWHAG, YWHAE, PRKCB |
| Erythropoietin Signaling | 5,35E-01 | 2,47E-02 | JUN, PRKCB |
| LPS-stimulated MAPK Signaling | 5,35E-01 | 2,44E-02 | JUN, PRKCB |
| Caveolar-mediated Endocytosis Signaling | 5,27E-01 | 2,41E-02 | ACTB, ITGB7 |
| Non-Small Cell Lung Cancer Signaling | 5,27E-01 | 2,44E-02 | TFDP1, FOXO3 |
| Tumoricidal Function of Hepatic Natural Killer Cells | 5,24E-01 | 4,17E-02 | CYCS (includes EG:54205) |
| ERK/MAPK Signaling | 5,24E-01 | 1,96E-02 | MYCN, YWHAG, STAT3, PRKCB |
| Antiproliferative Role of Somatostatin Receptor 2 | 5,2E-01 | 2,53E-02 | ADCY4, GNG10 |
| Chemokine Signaling | 5,2E-01 | 2,67E-02 | JUN, PRKCB |
| β-alanine Metabolism | 5,2E-01 | 2,11E-02 | SMS, ALDH5A1 |
| Protein Kinase A Signaling | 5,18E-01 | 1,88E-02 | YWHAG, YWHAE, ADCY4, PDE1B, GNG10, PRKCB |
| Integrin Signaling | 5,14E-01 | 1,99E-02 | ACTR2, ARF3, ACTB, ITGB7 |
| Insulin Receptor Signaling | 5,13E-01 | 2,21E-02 | SGK1, FOXO3, ACLY |
| Small Cell Lung Cancer Signaling | 5,04E-01 | 2,2E-02 | TFDP1, CYCS (includes EG:54205) |
| HER-2 Signaling in Breast Cancer | 4,97E-01 | 2,44E-02 | ITGB7, PRKCB |
| PI3K Signaling in B Lymphocytes | 4,96E-01 | 2,11E-02 | JUN, FOXO3, PRKCB |
| Antiproliferative Role of TOB in T Cell Signaling | 4,95E-01 | 3,85E-02 | CCNA2 |
| VDR/RXR Activation | 4,89E-01 | 2,47E-02 | CSNK2A1, PRKCB |
| Leptin Signaling in Obesity | 4,89E-01 | 2,38E-02 | ADCY4, STAT3 |
| Cardiac β-adrenergic Signaling | 4,85E-01 | 2E-02 | ADCY4, PDE1B, GNG10 |
| TNFR2 Signaling | 4,69E-01 | 3,03E-02 | JUN |
| Bile Acid Biosynthesis | 4,68E-01 | 1,96E-02 | DHRS2 (includes EG:10202), ALDH5A1 |
| Cellular Effects of Sildenafil (Viagra) | 4,64E-01 | 2,04E-02 | ADCY4, ACTB, PDE1B |
| Aminoacyl-tRNA Biosynthesis | 4,62E-01 | 2,38E-02 | MARS2, IARS |
| 4-1BB Signaling in T Lymphocytes | 4,57E-01 | 2,94E-02 | JUN |
| Hepatic Cholestasis | 4,54E-01 | 1,76E-02 | JUN, ADCY4, PRKCB |
| Ceramide Signaling | 4,48E-01 | 2,22E-02 | JUN, CYCS (includes EG:54205) |
| Sonic Hedgehog Signaling | 4,45E-01 | 3,03E-02 | CDK1 |
| Regulation of Actin-based Motility by Rho | 4,42E-01 | 2,2E-02 | ACTR2, ACTB |
| Prostate Cancer Signaling | 4,42E-01 | 2,04E-02 | TFDP1, HSP90AB1 |
| EIF2 Signaling | 4,36E-01 | 1,96E-02 | EIF5, PPP1R15A |
| Phenylalanine, Tyrosine and Tryptophan Biosynthesis | 4,34E-01 | 1,52E-02 | ENO3 |
| Ascorbate and Aldarate Metabolism | 4,34E-01 | 1,25E-02 | ALDH5A1 |
| mTOR Signaling | 4,3E-01 | 1,89E-02 | HMOX1, EIF4B, PRKCB |
| B Cell Receptor Signaling | 4,21E-01 | 1,91E-02 | JUN, MAP3K8, PRKCB |
| TWEAK Signaling | 4,12E-01 | 2,63E-02 | CYCS (includes EG:54205) |
| Fatty Acid Metabolism | 4,12E-01 | 1,78E-02 | ACAT2, DHRS2 (includes EG:10202), ALDH5A1 |
| CDK5 Signaling | 4,11E-01 | 2,13E-02 | FOSB, ADCY4 |
| PPAR Signaling | 4,11E-01 | 1,96E-02 | JUN, HSP90AB1 |
| Crosstalk between Dendritic Cells and Natural Killer Cells | 4,06E-01 | 2,06E-02 | ACTB, LTB |
| RANK Signaling in Osteoclasts | 4,06E-01 | 2,02E-02 | JUN, MAP3K8 |
| Pyruvate Metabolism | 4,06E-01 | 1,42E-02 | ACAT2, ALDH5A1 |
| Propanoate Metabolism | 4,06E-01 | 1,56E-02 | ACAT2, ALDH5A1 |
| Aminosugars Metabolism | 4,06E-01 | 1,63E-02 | PDE1B, RENBP |
| Tight Junction Signaling | 4,03E-01 | 1,85E-02 | JUN, NUDT21, ACTB |
| Oxidative Phosphorylation | 4,03E-01 | 1,94E-02 | SDHA, ATP5C1, ATP5F1 |
| IL-9 Signaling | 4,02E-01 | 2,5E-02 | STAT3 |
| Oncostatin M Signaling | 4,02E-01 | 2,86E-02 | STAT3 |
| G Protein Signaling Mediated by Tubby | 4,02E-01 | 2,56E-02 | GNG10 |
| Interferon Signaling | 4,02E-01 | 2,78E-02 | STAT2 |
| Neuregulin Signaling | 3,94E-01 | 1,94E-02 | HSP90AB1, PRKCB |
| SAPK/JNK Signaling | 3,94E-01 | 1,98E-02 | JUN, HNRNPK |
| Nucleotide Excision Repair Pathway | 3,93E-01 | 2,86E-02 | POLR2B |
| Folate Biosynthesis | 3,93E-01 | 1,33E-02 | DHFR |
| Riboflavin Metabolism | 3,93E-01 | 1,89E-02 | ACP1 |
| Chronic Myeloid Leukemia Signaling | 3,89E-01 | 1,89E-02 | CTBP1, TFDP1 |
| Inhibition of Angiogenesis by TSP1 | 3,83E-01 | 2,78E-02 | JUN |
| April Mediated Signaling | 3,66E-01 | 2,33E-02 | JUN |
| Production of Nitric Oxide and Reactive Oxygen Species in Macrophages | 3,62E-01 | 1,59E-02 | JUN, MAP3K8, PRKCB |
| Cardiac Hypertrophy Signaling | 3,54E-01 | 1,65E-02 | JUN, ADCY4, MAP3K8, GNG10 |
| G Beta Gamma Signaling | 3,53E-01 | 1,72E-02 | GNG10, PRKCB |
| Role of PKR in Interferon Induction and Antiviral Response | 3,49E-01 | 2,22E-02 | CYCS (includes EG:54205) |
| B Cell Activating Factor Signaling | 3,49E-01 | 2,22E-02 | JUN |
| Pantothenate and CoA Biosynthesis | 3,49E-01 | 1,59E-02 | BCAT1 |
| Glioma Signaling | 3,43E-01 | 1,72E-02 | TFDP1, PRKCB |
| Phospholipase C Signaling | 3,42E-01 | 1,56E-02 | HMOX1, ADCY4, GNG10, PRKCB |
| Aminophosphonate Metabolism | 3,41E-01 | 1,54E-02 | CHPT1 |
| Wnt/β-catenin Signaling | 3,36E-01 | 1,72E-02 | JUN, CSNK2A1, DVL3 |
| Cholecystokinin/Gastrin-mediated Signaling | 3,34E-01 | 1,89E-02 | JUN, PRKCB |
| Rac Signaling | 3,29E-01 | 1,63E-02 | ACTR2, JUN |
| Colorectal Cancer Metastasis Signaling | 3,27E-01 | 1,57E-02 | JUN, ADCY4, STAT3, GNG10 |
| MIF Regulation of Innate Immunity | 3,26E-01 | 2,04E-02 | JUN |
| IL-8 Signaling | 3,22E-01 | 1,57E-02 | HMOX1, GNG10, PRKCB |
| PTEN Signaling | 3,21E-01 | 1,63E-02 | FOXO3, CSNK2A1 |
| Primary Immunodeficiency Signaling | 3,06E-01 | 1,82E-02 | IGLL1 |
| Neuroprotective Role of THOP1 in Alzheimer's Disease | 3,06E-01 | 1,85E-02 | YWHAE |
| MSP-RON Signaling Pathway | 3,06E-01 | 2E-02 | ACTB |
| Sphingosine-1-phosphate Signaling | 3,04E-01 | 1,67E-02 | ADCY4, CASP2 |
| Glycosphingolipid Biosynthesis - Ganglioseries | 2,99E-01 | 1,61E-02 | ELOVL6 |
| Docosahexaenoic Acid (DHA) Signaling | 2,99E-01 | 1,96E-02 | CYCS (includes EG:54205) |
| G-Protein Coupled Receptor Signaling | 2,98E-01 | 1,51E-02 | RGS2, GPER, LPAR2, ADCY4, PDE1B, MAP3K8, STAT3, PRKCB |
| PKCθ Signaling in T Lymphocytes | 2,88E-01 | 1,47E-02 | JUN, MAP3K8 |
| Cytotoxic T Lymphocyte-mediated Apoptosis of Target Cells | 2,87E-01 | 1,89E-02 | CYCS (includes EG:54205) |
| CNTF Signaling | 2,87E-01 | 1,92E-02 | STAT3 |
| Toll-like Receptor Signaling | 2,87E-01 | 1,82E-02 | JUN |
| Glycerolipid Metabolism | 2,84E-01 | 1,32E-02 | DHRS2 (includes EG:10202), ALDH5A1 |
| Assembly of RNA Polymerase II Complex | 2,81E-01 | 1,79E-02 | POLR2B |
| Role of NFAT in Cardiac Hypertrophy | 2,8E-01 | 1,47E-02 | ADCY4, GNG10, PRKCB |
| Gα12/13 Signaling | 2,8E-01 | 1,57E-02 | JUN, LPAR2 |
| CCR3 Signaling in Eosinophils | 2,76E-01 | 1,57E-02 | GNG10, PRKCB |
| GABA Receptor Signaling | 2,69E-01 | 1,79E-02 | ALDH5A1 |
| CD28 Signaling in T Helper Cells | 2,69E-01 | 1,5E-02 | ACTR2, JUN |
| Tyrosine Metabolism | 2,62E-01 | 9,95E-03 | DHRS2 (includes EG:10202), ELOVL6 |
| Thrombin Signaling | 2,6E-01 | 1,46E-02 | ADCY4, GNG10, PRKCB |
| Nur77 Signaling in T Lymphocytes | 2,59E-01 | 1,64E-02 | CYCS (includes EG:54205) |
| OX40 Signaling Pathway | 2,59E-01 | 1,59E-02 | JUN |
| Cell Cycle: G1/S Checkpoint Regulation | 2,59E-01 | 1,69E-02 | TFDP1 |
| Amyloid Processing | 2,59E-01 | 1,75E-02 | CSNK2A1 |
| Phenylalanine Metabolism | 2,59E-01 | 9,35E-03 | ELOVL6 |
| TREM1 Signaling | 2,53E-01 | 1,52E-02 | STAT3 |
| Estrogen Receptor Signaling | 2,49E-01 | 1,46E-02 | CTBP1, POLR2B |
| Endometrial Cancer Signaling | 2,44E-01 | 1,67E-02 | FOXO3 |
| Cdc42 Signaling | 2,42E-01 | 1,38E-02 | ACTR2, JUN |
| Arginine and Proline Metabolism | 2,39E-01 | 1,1E-02 | SMS, ALDH5A1 |
| Pentose Phosphate Pathway | 2,39E-01 | 1,19E-02 | PRPS1 |
| Induction of Apoptosis by HIV1 | 2,34E-01 | 1,56E-02 | CYCS (includes EG:54205) |
| Urea Cycle and Metabolism of Amino Groups | 2,25E-01 | 1,25E-02 | SMS |
| Retinoic acid Mediated Apoptosis Signaling | 2,17E-01 | 1,54E-02 | CYCS (includes EG:54205) |
| Calcium-induced T Lymphocyte Apoptosis | 2,12E-01 | 1,47E-02 | PRKCB |
| IL-15 Signaling | 2,12E-01 | 1,43E-02 | STAT3 |
| Glutamate Metabolism | 2,08E-01 | 1,3E-02 | ALDH5A1 |
| Estrogen-Dependent Breast Cancer Signaling | 2,01E-01 | 1,37E-02 | JUN |

E-x: 10^x^

**S4.** Enzymatic activity of caspase 3/7 after 6 h treatment of CCRF-CEM cells. The activity of caspase 3/7 is expressed as percentage % relative to untreated cells.

**S5.** Top 10 signaling pathways affected by guieranone A treatment in CCRF-CEM cells. The evaluation of differentially expressed genes was performed using the Ingenuity Pathway Analysis software. (List of all pathways in supplemental data 9)


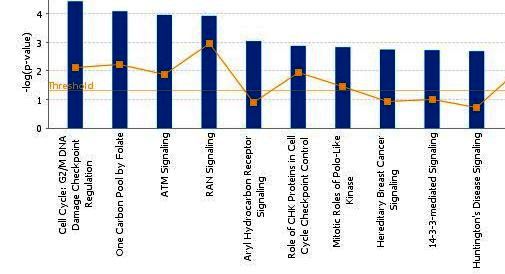


**S6.** Results of real-time reverse transcriptase PCR analysis. CCRF-CEM cells were treated with IC­_50_ concentration of guieranone for 24 h, Transcriptional changes are expressed relative to G6PD. The mean value ±SEM of three independent experiments is shown.


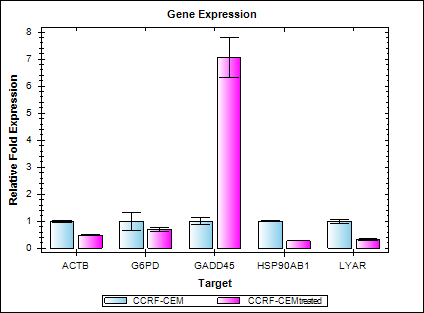


**Table S7.**

Functions associated with the networks for genes whose expression was affected

by treatment with guieranone A

| ID | Score | Focus Molecules | Top Functions |
| --- | --- | --- | --- |
| 1 | 50 | 28 | Molecular Transport, Protein Trafficking, Cellular Assembly and Organization |
| 2 | 50 | 28 | Drug Metabolism, Endocrine System Development and Function, Lipid Metabolism |
| 3 | 47 | 27 | Cell Death, Cell-To-Cell Signaling and Interaction, Cellular Function and Maintenance |
| 4 | 45 | 26 | Cell Cycle, Cancer, Gastrointestinal Disease |
| 5 | 33 | 21 | Cell-To-Cell Signaling and Interaction, Gene Expression, Cell Death |
| 6 | 24 | 17 | Molecular Transport, Nucleic Acid Metabolism, Small Molecule Biochemistry |
| 7 | 24 | 17 | Cellular Development, Cell Cycle, Cellular Growth and Proliferation |
| 8 | 21 | 15 | Drug Metabolism, Cellular Compromise, Embryonic Development |
| 9 | 19 | 14 | Skeletal and Muscular Disorders, Embryonic Development, Tissue Morphology |
| 10 | 19 | 14 | Dermatological Diseases and Conditions, Immunological Disease, Inflammatory Disease |
| 11 | 19 | 14 | Small Molecule Biochemistry, Cellular Development, Cellular Growth and Proliferation |
| 12 | 17 | 13 | Energy Production, Nucleic Acid Metabolism, Small Molecule Biochemistry |
| 13 | 14 | 12 | Cellular Function and Maintenance, Cell Signaling, Vitamin and Mineral Metabolism |
| 14 | 12 | 10 | Cellular Development, Cellular Growth and Proliferation, Embryonic Development |
| 15 | 7 | 7 | Cell-To-Cell Signaling and Interaction, Cellular Assembly and Organization, Cell Signaling |
| 16 | 2 | 1 | Gene Expression |
| 17 | 2 | 1 | Embryonic Development, Tissue Morphology, Cellular Development |
| 18 | 2 | 1 | Cancer, Genetic Disorder, Reproductive System Disease |
| 19 | 2 | 1 | Carbohydrate Metabolism, Cellular Compromise, Embryonic Development |
| 20 | 2 | 1 | Molecular Transport, Genetic Disorder, Neurological Disease |
| 21 | 2 | 1 | Cellular Assembly and Organization, Cellular Function and Maintenance |
| 23 | 2 | 1 | Carbohydrate Metabolism, Small Molecule Biochemistry, Lipid Metabolism |
| 24 | 2 | 1 | Carbohydrate Metabolism, Lipid Metabolism, Small Molecule Biochemistry |
